# Deletion of BMP receptor type IB decreased bone mass in association with compromised osteoblastic differentiation of bone marrow mesenchymal progenitors

**DOI:** 10.1038/srep24256

**Published:** 2016-04-06

**Authors:** Ce Shi, Ayaka Iura, Masahiko Terajima, Fei Liu, Karen Lyons, Haichun Pan, Honghao Zhang, Mitsuo Yamauchi, Yuji Mishina, Hongchen Sun

**Affiliations:** 1 Department of Oral Pathology, School and Hospital of Stomatology, Jilin University, Changchun, 130021, China; 2 Department of Biologic and Materials Sciences, University of Michigan, School of Dentistry, Ann Arbor, MI 48109-1078, USA; 3 School of Dentistry, University of North Carolina, Chapel Hill, NC 27514, USA; 4 Department of Molecular, Cell and Developmental Biology, University of California, Los Angeles, Los Angeles, CA 90095, USA; 5 Department of Orthopaedic Surgery, University of California, Los Angeles, CA 90095, USA; 6 Orthopaedic Institute for Children, Los Angeles, CA 90007, USA

## Abstract

We previously found that disruption of two type I BMP receptors, *Bmpr1a* and *Acvr1*, respectively, in an osteoblast-specific manner, increased bone mass in mice. BMPR1B, another BMP type I receptor, is also capable of binding to BMP ligands and transduce BMP signaling. However, little is known about the function of BMPR1B in bone. In this study, we investigated the bone phenotype in *Bmpr1b* null mice and the impacts of loss of *Bmpr1b* on osteoblasts and osteoclasts. We found that deletion of *Bmpr1b* resulted in osteopenia in 8-week-old male mice, and the phenotype was transient and gender specific. The decreased bone mass was neither due to the changes in osteoblastic bone formation activity nor osteoclastic bone resorption activity *in vivo. In vitro* differentiation of *Bmpr1b* null osteoclasts was increased but resorption activity was decreased. Calvarial pre-osteoblasts from *Bmpr1b* mutant showed comparable differentiation capability *in vitro*, while they showed increased BMP-SMAD signaling in culture. Different from calvarial pre-osteoblasts, *Bmpr1b* mutant bone marrow mesenchymal progenitors showed compromised differentiation *in vitro*, which may be a reason for the osteopenic phenotype in the mutant mice. In conclusion, our results suggested that BMPR1B plays distinct roles from BMPR1A and ACVR1 in maintaining bone mass and transducing BMP signaling.

Bone morphogenetic proteins (BMPs) were originally identified as factors that can induce the formation of bone and cartilage when implanted at ectopic sites^1^. Recombinant human BMPs, rhBMP-2 and rhBMP-7, have been approved by US Food and Drug Administration (FDA) for clinical use of a variety of bone related conditions, including non-union, open fractures, joint fusions, aseptic bone necrosis and critical bone defects^2^. However, how BMP regulates bone homeostasis, the variable effects of BMP signaling on different cell types in bone, and how BMP signaling is transduced through different BMP receptors, are not fully elucidated yet. Thus, the answers to these questions may increase the efficacy of BMP therapeutics and provide new targets of treating bone related diseases.

BMPs are members of the transforming growth factor-β (TGF-β) superfamily. The TGF-β superfamily proteins bind to two types of serine/threonine kinase receptors, i.e. type I and type II receptors^3^. Both type I and type II receptors are required for signal transduction^4^. Among various type I receptors, ACVR1 (activin receptor type I, also known as activin receptor-like kinase 2, ALK2), BMPR1A (BMP receptor type IA, also known as ALK3) and BMPR1B (BMP receptor type IB, also known as ALK6) are capable of mediating BMP ligand signal. BMP type I receptors are transphosphorylated by BMP type II receptors upon ligand binding, and then phosphorylate the cytoplasmic signaling molecules to initiate SMAD and non-SMAD signaling cascades^5^.

Previously, we reported that an osteoblast-specific deletion of *Bmpr1a* or *Acvr1* results in increased bone mass^6^,^7^,^8^,^9^,^10^. Both BMPR1A and ACVR1 in osteoblasts influenced bone modeling and bone remodeling through up-regulation of *Sost* and *Dkk1*, resulted in the suppression of canonical WNT pathway and the increase of RANKL/OPG ratio. Thus the disruption of *Bmpr1a* or *Acvr1* breaks the balance of bone formation and bone resorption, i.e. slightly decreased osteoblast activity and dramatically decreased osteoclast activity. BMPR1B, another BMP type I receptor, is also capable of binding to BMP ligands and transduce BMP signaling. Unlike *Bmpr1a* or *Acvr1* homozygous null mice that show early embryonic lethality^11^,^12^, *Bmpr1b* homozygous null mice are viable exhibiting forelimb and hindlimb defects, due to markedly reduced proliferation of prechondrogenic cells and chondrocyte differentiation in the phalangeal region^13^,^14^. However, the bone phenotype of *Bmpr1b* null mice and the mechanisms of how BMPR1B regulates the activity of osteoblasts and osteoclasts have not been studied yet.

In this study, we investigated the bone phenotype of *Bmpr1b* null mice *in vivo* and the effects of *Bmpr1b* deletion on osteoclasts and osteoblastic lineage cells *in vitro*. We found that deletion of *Bmpr1b* leads to osteopenia in 8-week-old male mice, which is likely due to the compromised osteogenic differentiation of bone marrow mesenchymal progenitors, but not that of pre-osteoblasts. In addition, we compared BMP-SMAD and non-SMAD signaling mediated by different BMP type I receptors and found that deletion of *Bmpr1b* sensitized BMP-SMAD signaling in cultured calvarial pre-osteoblasts, which was different from the other two BMP type I receptors. We identified that BMPR1B plays distinct roles in maintaining bone homeostasis and transducing BMP signaling compared with ACVR1 and BMPR1A.

## Results

### *Bmpr1b* deletion leads to lowered bone mass in mice

*Bmpr1b* homozygous mutant mice (*Bmpr1b*^−/−^, KO) and littermate controls (*Bmpr1b*^+/+^, WT and *Bmpr1b*^+/−^, Het) were born with a normal Mendelian ratio (Supplementary Table S1). At newborn stage, no overt histologic differences were found in femur from either males or females (Supplementary Fig. S1). At 8 weeks old, KO males weighed slightly but significantly less (10%) (p < 0.05) than littermate WT males (Supplementary Fig. S2). However, 8-week-old females had similar body weight between WT and KO (Supplementary Fig. S2).

**Figure 1:**
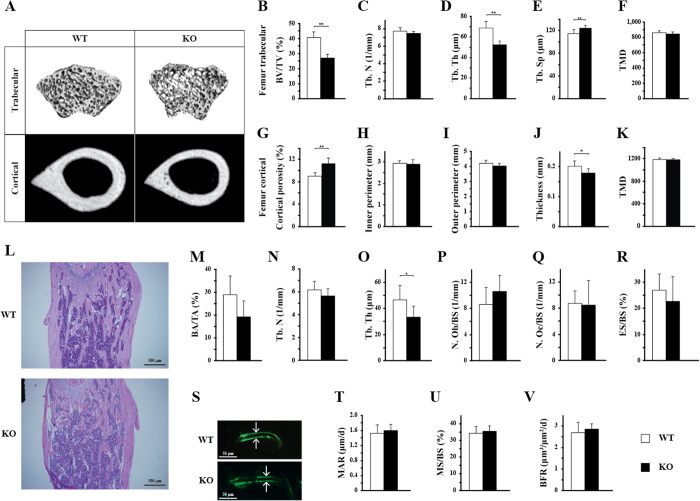
*Bmpr1b* deletion leads to osteopenia in 8-week-old male mice, but does not change osteoblastic bone formation activity or osteoclastic bone resorption activity *in vivo*. (**A**) Representative micro-CT images of trabecular compartment of distal femur and cortical compartment of middle femur from 8-week-old males. (**B–F**) Trabecular parameters were determined by micro-CT for the femora from 8-week-old male mice: (**B**) trabecular bone volume fraction (bone volume/tissue volume, BV/TV); (**C**) trabecular number (Tb. N); (**D**) trabecular thickness (Tb.Th); (**E**) trabecular separation (Tb. Sp); (**F**) tissue mineral density (TMD); (**G–K**) Cortical parameters: (**G**) cortical porosity; (**H**) inner perimeter; (**I**) outer perimeter; (**J**) cortical thickness; (**K**) TMD. For each group, n = 8. (**L–R**) Static histomorphometry for the trabecular compartment of distal femora of 8-week-old male mice (for each group, n = 6): (**L**) Representative H&E stained sections; (**M**) trabecular bone area/tissue area (BA/TA); (**N**) trabecular bone number (Tb. N); (**O**) trabecular bone thickness (Tb. Th); (**P**) osteoblast number per bone surface (N. Ob/BS); (**Q**) osteoclast number per bone surface (N. Oc/BS); (**R**) eroded surface per bone surface (ES/BS); (**S**) Representative calcein labeling images (5 days apart between two labels). (**T–V**) Dynamic histomorphometry for the femora of 8-week-old male mice (for each group, n = 8): (**T**) mineral apposition rate (MAR); (**U**) mineralized surface per bone surface (MS/BS); (**V**) bone formation rate (BFR/BS). *p < 0.05; **p < 0.01. WT: wild type; KO: knockout.

Micro-CT results showed an osteopenic phenotype in 8-week-old KO male mice (Fig. 1A). In femur trabecular compartment of KO mice (Fig. 1B–F and Supplementary Fig. S3A,B), we observed a 34% decrease in trabecular bone volume fraction (BV/TV) (p < 0.001), a 24% decrease in trabecular thickness (Tb. Th) (p < 0.001), a slight increase (8%) in trabecular separation (Tb. Sp) (p < 0.01), and a 23% decrease in bone mineral density (BMD) (p < 0.001), but no difference in tissue mineral density (TMD) (p > 0.05). In femur cortical compartment of KO mice (Fig. 1G–K and Supplementary Fig. S3C–E), we observed a 25% increase in cortical porosity (1-BV/TV) (p < 0.001), a 4% decrease in outer perimeter (p = 0.08), a 11% decrease in cortical thickness (p < 0.05), a slight decrease in cortical bone volume fraction (BV/TV) (2.5%) (p < 0.001), a 7% decrease in bone mineral density (BMD) (p < 0.05), but no difference in tissue mineral density (TMD) (p > 0.05), and a 13% decrease in cortical area (p < 0.05). However, these differences were not seen in 8-week-old females (Supplementary Fig. S4). In 11-week-old males (Supplementary Fig. S5), although they showed a 25% decrease in trabecular thickness (Tb. Th) by static histomorphometry (p < 0.05) (Supplementary Fig. S5R), we did not detect overt differences in all trabecular parameters investigated by micro-CT. In the cortical compartment, we detected a slight decrease (8%) in bone volume fraction (BV/TV) and a 63% increase in cortical porosity (1-BV/TV) (p < 0.05) by microCT (Supplementary Fig. S5G,H). These results suggested that the bone phenotypes observed in *Bmpr1b* mutant mice are gender specific and transient.

### Disruption of *Bmpr1b* does not change osteoblastic bone formation activity or osteoclastic bone resorption activity in mice

Next, we performed static (Fig. 1M–R and Supplementary Fig. S3F–H) and dynamic (Fig. 1S–V) histomorphometry on distal femora. Using H&E stained sections of 8-week-old samples (Fig. 1L), we identified a 28% reduction of trabecular thickness (Tb. Th) (Fig. 1O, p < 0.05) as consistent with micro-CT data. However, we did not observe overt changes in the other indices of static histomorphometry (Fig. 1M–R and Supplementary Fig. S3F–H). We noticed a decreased tendency of bone area (BA/TA) (Fig. 1M, p = 0.06) and an increased tendency of trabecular bone separation (Tb. Sp) (Supplementary Fig. S3F, p = 0.09), which are also consistent with micro-CT measurements (Fig. 1B,E). In addition, for dynamic histomorphometry (Fig. 1S–V), there was no difference between WT and KO with regard to mineral apposition rate (MAR), mineralized surface (MS), or bone formation rate (BFR). Taken together, static and dynamic histomorphemetry suggested that the decreased bone mass observed in KO mice is not due to the changes in osteoblast number and bone formation function or osteoclast number and bone resorption function *in vivo*.

To determine serum biological markers for both bone formation and bone resorption, we measured levels of N-terminal propeptide of type 1 procollagen (P1NP), collagen type 1 cross-linked C-telopeptide (CTX-1), calcium and phosphorus in serum. We found there was no difference between WT and KO (Supplementary Fig. S6A–C).

Next, to determine osteogenic capability of osteoblasts *in vitro*, calvarial pre-osteoblasts were cultured in osteogenic medium. ALP and alizarin red staining showed no difference between WT and KO (Supplementary Fig. S7). P1NP level in culture medium was not changed (Supplementary Fig. S6D,E). In calvarial pre-osteoblasts treated with or without BMP, there was no difference in the expressions of *Bmpr1a* and *Acvr1* between WT and KO (Supplementary Fig. S8), suggesting that *Bmpr1b* deletion was not compensated by other type I receptors. Moreover, there was no difference in the expressions of osteoblast marker genes or genes related with BMP signaling in cultured calvarial pre-osteoblasts (Supplementary Fig. S9) or in tibiae from both males and females (Supplementary Fig. S10). Some genes that regulate WNT signaling show reductions in *Bmpr1b* KO calvarial pre-osteoblasts (*Sost* and *Dkk2* in Supplementary Fig. S9) and 8-week-old males (*Lrp5*, *Sost* and *Dkk1* in Supplementary Fig. S10), however, we did not see differences in the WNT target genes (*Axin2* and *Lef1*). These results suggested that deletion of *Bmpr1b* did not change the differentiation ability of calvarial pre-osteoblasts *in vitro*.

In terms of collagen content and structure both in tibiae and cultured calvarial pre-osteoblasts, there were no differences between WT and KO in collagen composition (Supplementary Fig. S11A,D), lysine hydroxylation (Supplementary Fig. S11B,E), or collagen cross-links (Supplementary Fig. S11C,F). In addition, there was no consistent difference between WT and KO in gene expression levels of collagen modifying enzymes (Supplementary Fig. S12). These results suggested that deletion of *Bmpr1b* did not change the collagen deposition by osteoblasts or the post-translational modifications of collagen both in- and out-side of osteoblasts.

**Figure 2:**
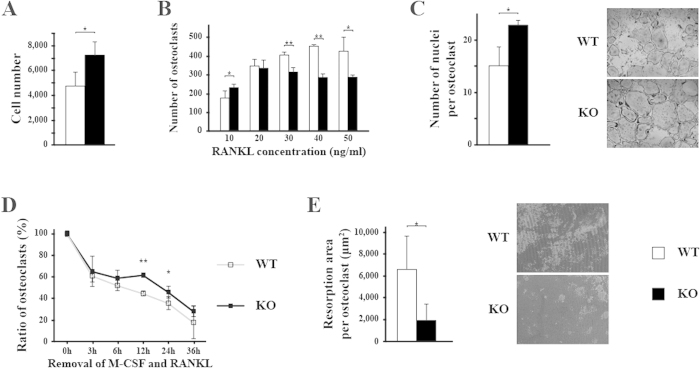
*In vitro* differentiation of osteoclasts from KO mice is increased but resorption activity is decreased. (**A**) Osteoclast precursors were treated with 30 ng/ml M-CSF for 6 days, and the number of cells was counted. (**B**) Osteoclasts precursors were prompted differentiation with gradient concentration of RANKL for 6 days. Cells were stained with TRAP, and the number of osteoclasts (**B**) as well as the number of nuclei per osteoclasts (**C**) were counted. (**C**) Representative images of osteoclast differentiation from WT and KO mice. (**D**) Osteoclasts precursors were differentiated with 30 ng/ml M-CSF and 50 ng/ml RANKL. On day 6, M-CSF and RANKL were removed from the culture, and number of osteoclasts was counted at indicated time points. The numbers of osteoclasts at each time point was normalized to that at time point 0. (**E**) Osteoclast precursors were cultured on coated surface and treated with 30 ng/ml M-CSF and 50 ng/ml RANKL. After 6 days, the area of resorption was measured and the number of osteoclasts on coated surface was counted. Resorption area was normalized by osteoclast number. Representative images of resorption by WT and KO osteoclasts. *p < 0.05; **p < 0.01. WT: wild type; KO: knockout.

To determine osteoclast activity *in vitro*, we investigated the proliferation and differentiation of osteoclast precursors, and survival and bone resorption function of mature osteoclasts. After osteoclast precursors were treated with M-CSF only, there were more cells in KO compared with WT (Fig. 2A), indicating that osteoclast precursors from KO mice either have a higher proliferation rate or a lower apoptosis. When treated with M-CSF and gradient concentrations of RANKL, WT and KO osteoclast precursors showed different responses to RANKL. When RANKL was at low concentration (10 ng/ml), there were more KO osteoclasts compared with WT. As RANKL concentration increased, there were fewer KO osteoclasts compared with WT (Fig. 2B). As shown in Fig. 2C, KO osteoclasts were much larger with more nuclei than WT osteoclasts, thus there were not enough spaces for more osteoclasts formation when RANKL concentration was increased. It suggested that KO osteoclast precursors were more sensitive to RANKL and had an increased fusion into multinucleated osteoclasts. After differentiation factors were removed, we observed there were more KO osteoclasts left compared with WT, especially at 12 h and 24 h after removal of the cytokines (Fig. 2D). These data suggested that BMP signaling through BMPR1B somehow affects osteoclast apoptosis. When osteoclasts were differentiated on inorganic hydroxyapatite coated surface, KO osteoclasts showed decreased resorption area as well as decreased resorption area per osteoclast (Fig. 2E). Taken together, *in vitro* results revealed increased number of osteoclast precursors, more osteoclast differentiation, longer survival and decreased resorption function from KO mice. However, these *in vitro* data were inconsistent with the *in vivo* static histomorphometry, which showed no difference in osteoclast number, osteoclast surface, or eroded surface (Fig. 1Q,R and Supplementary Fig. S3H) (See discussion). Taken together, these results suggested that the osteopenic phenotype observed in KO mice was not due to the changes in osteoblastic bone formation activity or osteoclastic bone resorption activity.

### Disruption of *Bmpr1b* alters BMP signaling pathway in calvarial pre-osteoblasts distinctly from disruption of *Bmpr1a* or *Acvr1*

**Figure 3:**
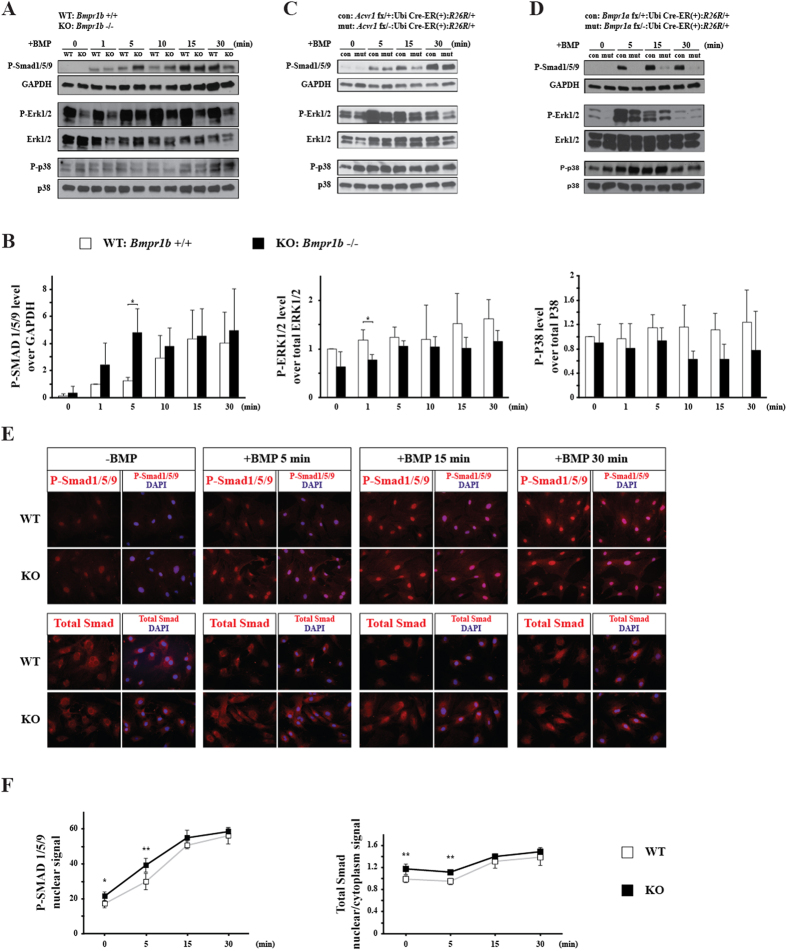
Deletion of *Bmpr1b* transduces BMP signaling pathway distinctly in calvarial pre-osteoblasts. (**A**) Cultured calvarial pre-osteoblasts from WT and KO were serum starved overnight, followed by rhBMP-2 stimulation for the indicated times. Cells were lysed and immunoblotted with P-SMAD1/5/9, P-ERK1/2 and P-P38. GAPDH, ERK1/2 and P38 were used as loading controls. (**B**) Quantification of the blotting of P-SMAD1/5/9, P-ERK1/2 and P-P38. (**C**) Cultured calvarial pre-osteoblasts from *Acvr1* fx/+ :Ubi Cre-ER^TM^ (+)/(−):*R26R*/+ (con) and *Acvr1* fx/-:Ubi Cre-ER^TM^ (+)/(−):*R26R*/+ (mut) were treated with TM and sorted as mentioned in materials and methods section. Cell lysate was immunoblotted as mentioned above. (**D**) Cultured calvarial pre-osteoblasts from *Bmpr1a* fx/+ :Ubi Cre-ER^TM^ (+)/(−):*R26R*/+ (con) and *Bmpr1a* fx/-:Ubi Cre-ER^TM^ (+)/(−):*R26R*/+ (mut) were treated with TM. Cell lysate was immunoblotted as mentioned above. (**E**) Cells were fixed and immunostained with P-SMAD1/5/9 and SMAD. (**F**) The mean density of P-SMAD1/5/9 signal in the nucleus and the signal ratio of nucleus and cytoplasm of total SMAD were quantified. *p < 0.05; **p < 0.01. WT: wild type; KO: knockout; con: control (fx/+ :Ubi Cre-ER(+)/(−):*R26R*/+); mut: mutant (fx/-:Ubi Cre-ER(+)/(−):*R26R*/+). For western blot results, the gels were run, and transferred to the membrane under the same experimental conditions, and the blots within comparisons were exposed at the same conditions. The blots were cropped. Full-length blots are presented in Supplementary Figure S13.

To investigate the regulation of BMP signaling mediated by BMP type I receptors, phosphorylation levels of SMAD1/5/9 (SMAD signaling) and ERK1/2 and P38 (non-SMAD signaling) in calvarial pre-osteoblasts were determined by western blot. Surprisingly, we found that deletion of *Bmpr1b* resulted in a higher level of p-SMAD1/5/9 at early time points upon BMP stimulation (1 min and 5 min), and the peak level of p-SMAD occurred earlier in KO cells, whereas P-ERK1/2 and P-P38 levels showed decreased tendency in KO cells compared with WT (Fig. 3A,B).

To determine whether the elevated P-SMAD1/5/9 can translocate into nucleus, we performed immunocytochemistry using calvarial pre-osteoblasts. The results showed that nuclear signal of P-SMAD1/5/9 was very weak without BMP stimulation, and total Smad was mainly distributed in the cytoplasm. After BMP stimulation, P-SMAD1/5/9 in both WT and KO cells can translocate into nucleus (Fig. 3E). At 5 min after BMP stimulation, the nuclear signal of P-SMAD1/5/9 was stronger in KO cells than that in WT (Fig. 3F), demonstrating that the elevated P-SMAD1/5/9 in *Bmpr1b* KO cells can translocate into nucleus. The questions that why P-SMAD level is higher in KO pre-osteoblasts and why higher level of BMP-SMAD signaling does not influence the expressions of osteoblast marker genes and osteoblast differentiation ability need to be further addressed.

We also examined the impacts of loss of other type I BMP receptors, ACVR1 and BMPR1A. In *Acvr1* KO calvarial pre-osteoblasts, P-SMAD1/5/9 level was slightly decreased, while there were no obvious changes in the levels of P-ERK1/2 and P-P38 (Fig. 3C). In *Bmpr1a* KO calvarial pre-osteoblasts, there was dramatic decrease in the levels of P-SMAD1/5/9 and P-ERK1/2, while there was no overt change in P-P38 level (Fig. 3D). Taken together, these results demonstrated that BMPR1B plays distinct roles from BMPR1A and ACVR1 in transducing BMP signaling in calvarial pre-osteoblasts.

### Osteoblastic differentiation of bone marrow mesenchymal progenitors is compromised in *Bmpr1b* KO mice

**Figure 4:**
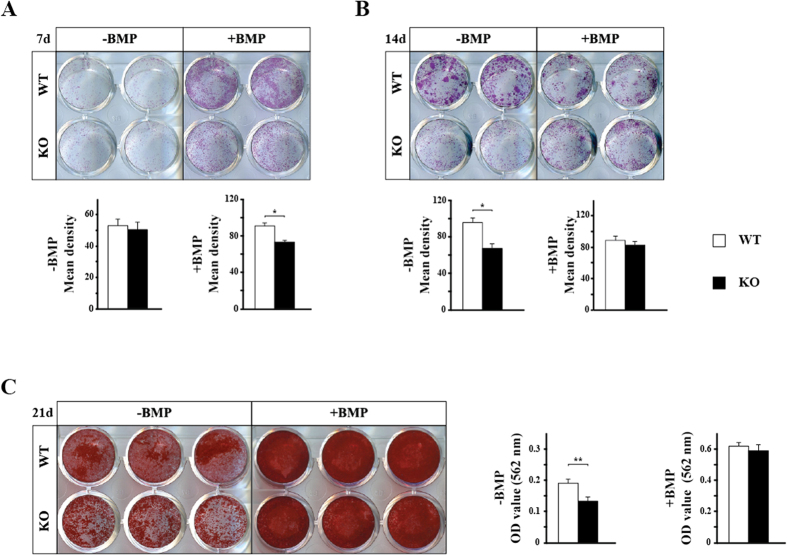
Osteoblastic differentiation of bone marrow mesenchymal progenitors is compromised in KO mice. Bone marrow mesenchymal progenitors were isolated from femora and tibiae of 8-week-old mice and subjected to osteoblast differentiation. Cells were treated with or without BMP-2. (**A**) Alkaline phosphatase (ALP) staining and mean density of the staining after 7 days of differentiation. (**B**) ALP staining and mean density of the staining after 14 days of differentiation. (**C**) Alizarin red staining and quantification of the staining after 21 days of differentiation. After staining, 300 μl/well of 10% CPC was used to dissolve bound alizarin red S. The dissolved solution was diluted 10 times with 10% CPC before measurement. *p < 0.05; **p < 0.01. WT: wild type; KO: knockout.

To further determine the reason why *Bmpr1b* KO mice show the osteopenic phenotype, we investigated the effect of *Bmpr1b* deletion on osteoblastic differentiation of bone marrow mesenchymal progenitors *in vitro*. Bone marrow cells from the KO mice showed significantly less ALP staining after differentiation was prompted by BMP at day 7 (Fig. 4A). At 14 days without BMP stimulation, KO cells showed significantly less ALP staining (Fig. 4B). Alizarin red staining pattern revealed that KO cells in the absence of BMP have less mineralized nodules, while there was no difference after stimulation of differentiation using BMP (Fig. 4C). Different from calvarial pre-osteoblasts, these results suggested that deletion of *Bmpr1b* compromised osteoblastic differentiation of bone marrow mesenchymal progenitors, which may be one of the potential reasons leading to osteopenic phenotype.

**Figure 5:**
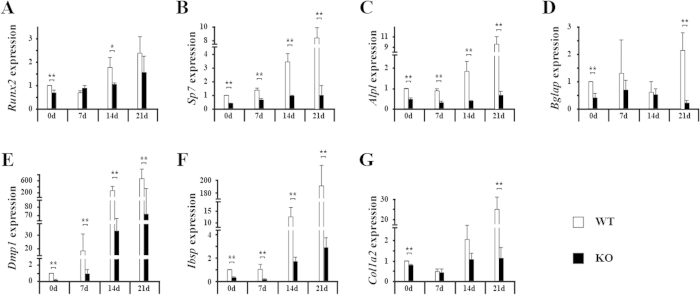
Expression of osteoblast marker genes was decreased in KO bone marrow mesenchymal progenitors. Bone marrow cells were cultured in osteogenic medium for the indicated days. Total mRNA was isolated, and quantitative RT-PCR was performed. Expression of *Runx2* (**A**), *Sp7* (**B**), *Alp1* (**C**), *Ocn* (**D**), *Dmp1* (**E**), *Ibsp* (**F**) and *Col1a2* (**G**) was calculated. *p < 0.05; **p < 0.01. WT: wild type; KO: knockout.

**Figure 6:**
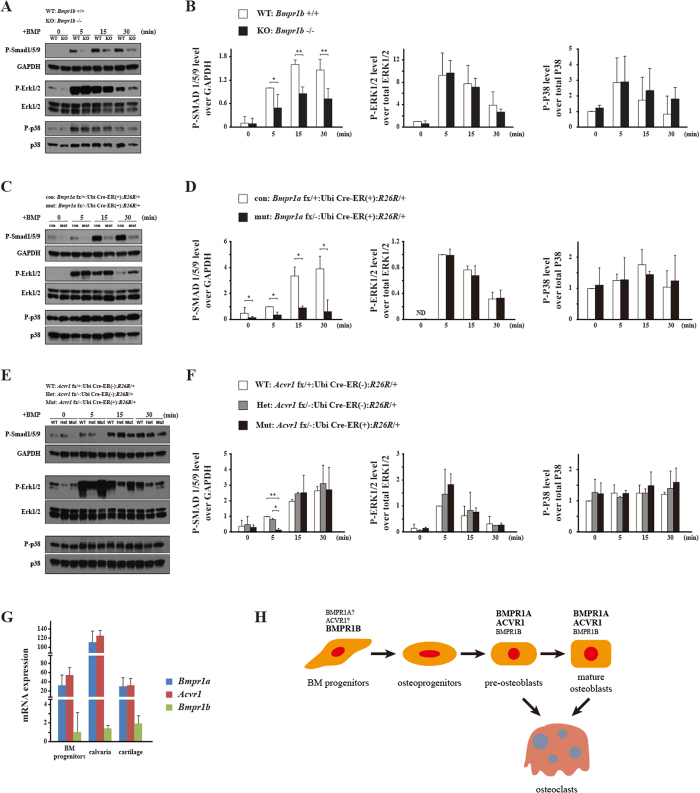
BMP signaling was decreased in bone marrow mesenchymal progenitors deficient for *Bmpr1b* (**A,B**), *Bmpr1a* (**C,D**) and *Acvr1* (**E,F**), respectively. (**A,C,E**) Bone marrow mesenchymal progenitors were serum starved overnight, followed by rhBMP-2 stimulation for the indicated times. Cells were lysed and immunoblotted with P-SMAD1/5/9, P-ERK1/2 and P-P38. GAPDH, ERK1/2 and P38 were used as loading controls. The gels were run, and transferred to the membrane under the same experimental conditions and the blots within comparisons were exposed at the same conditions. The blots were cropped. Full-length blots are presented in Supplementary Fig. S13. (**B,D,F**) Quantification of the blotting of P-SMAD1/5/9, P-ERK1/2 and P-P38. (**G**) Expression pattern of *Bmpr1a*, *Acvr1* and *Bmpr1b* in bone marrow mesenchymal progenitors, calvaria and cartilage. (**H**) A working model showing distinct roles of BMP type I receptors during osteoblastic differentiation from bone marrow mesenchymal progenitors. Our previous findings demonstrated that BMPR1A and ACVR1 in osteoblasts positively regulate osteoblast activity and osteoclast activity. Our current study found that BMPR1B positively regulate early osteoblastic differentiation of bone marrow mesenchymal progenitors, but not later stage. However, we still do not know how BMPR1A and ACVR1 regulate BMSCs, due to lack of mouse model targeting BMPR1A and ACVR1 in BMSCs. *p < 0.05; **p < 0.01. WT: wild type; KO: knockout; con: control (fx/+ :Ubi Cre-ER(+)/(−):*R26R*/+); mut: mutant (fx/-:Ubi Cre-ER(+)/(−):*R26R*/+); Het: heterozygous (fx/−:Ubi Cre-ER(−)/(−):*R26R*/+); BM: bone marrow mesenchymal.

To obtain the molecular insight of osteoblastic differentiation of bone marrow mesenchymal progenitors, gene expression pattern and BMP signaling were examined. Quantitative RT-PCR results showed that in bone marrow mesenchymal progenitors, the expressions of *Runx2*, *Sp7*, *Alpl*, *Bglap*, *Dmp1*, *Ibsp* and *Col1a2* during the culture period were dramatically decreased in *Bmpr1b* KO cells compared with WT cells (Fig. 5A–G). Different from calvarial pre-osteoblasts, deletion of *Bmpr1b* in bone marrow mesenchymal progenitors showed significant decreased P-SMAD1/5/9 in KO cells, compared with WT, while no overt changes in P-ERK1/2 or P-P38 levels (Fig. 6A,B). We also examined the impacts of loss of other BMP type I receptors in bone marrow mesenchymal progenitors. In *Bmpr1a* KO bone marrow mesenchymal progenitors, there was a dramatic decrease in the level of P-SMAD1/5/9, whereas there were no overt changes in the levels of P-ERK1/2 and P-P38 (Fig. 6C,D). In *Acvr1* KO bone marrow mesenchymal progenitors, there was a slight decrease in the level of P-SMAD1/5/9 at early time points upon BMP stimulation, whereas there were no overt changes in the levels of P-ERK1/2 and P-P38 (Fig. 6E,F).

To examine the expression pattern of the three BMP type I receptors in different types of cells or tissues, quantitative RT-PCR was performed. We found the expression levels of *Bmpr1b* were similar among different cells types, but much lower compared with *Acvr1* or *Bmpr1a* (Fig. 6G). However, the expression levels of *Bmpr1a* and *Acvr1* are more than 3 folds lower in bone marrow mesenchymal progenitors than those in calvaria, leading to a significantly higher ratio of *Bmpr1b* in bone marrow mesenchymal progenitors (Fig. 6G). These results suggested that deletion of *Bmpr1b* may affect more in behavior of bone marrow mesenchymal progenitors, but not calvarial pre-osteoblasts.

Taken together, these results indicated that different from calvarial pre-osteoblasts, osteoblastic differentiation of bone marrow mesenchymal progenitors from *Bmpr1b* KO mice was compromised, which may be due to the less capability to transduce BMP signaling and the subsequent gene transcription.

## Discussion

Our present results demonstrated that deletion of *Bmpr1b* leads to osteopenia in 8-week-old male mice. The osteopenic phenotype may be due to the compromised osteoblastic differentiation of bone marrow mesenchymal progenitors. Deletion of *Bmpr1b* did not change osteoclast behavior *in vivo*, while *in vitro* differentiation of *Bmpr1b* KO osteoclast was increased but resorption activity was decreased. In addition, deletion of *Bmpr1b* resulted in increased phosphorylation level of SMAD1/5/9 and its nuclear translocation at early time points upon BMP stimulation in calvarial pre-osteoblasts. Our results suggested that BMPR1B plays distinct roles in maintaining bone homeostasis and transducing BMP signaling. Based on our previous and current findings, we proposed a working model (Fig. 6H), showing distinct roles of BMP type I receptors during osteoblastic differentiation from bone marrow mesenchymal progenitors.

Although BMPR1B and BMPR1A share a high degree of sequence similarity, and have overlapping functions in chondrogenesis^15^, several studies indicated that they may possess distinct biological functions^16^,^17^,^18^. Our group showed that deletion of *Bmpr1a* using *Og2*-Cre show alterations in bone remodeling in an age-dependent manner^6^. We also found that deletion of *Bmpr1a* using a 3.2-kb *Col1*-CreER increases bone mass at both postnatal stage (3- and 22-week-old)^8^,^9^ and embryonic stage^7^, due to decreased osteoblast activity and more dramatically decreased osteoclast activity. BMPR1A in osteoblasts influenced bone modeling and bone remodeling through up-regulation of *Sost* and *Dkk1*, resulted in the suppression of canonical WNT pathway and the increase of RANKL/OPG ratio. Similarly, deletion of *Acvr1* using a 3.2-kb *Col1*-CreER increased bone mass at an embryonic stage (E18.5) and adult stage (3-week-old) in association with increased canonical WNT signaling via suppression of *Sost* and *Dkk1*^10^. Taken together, disruption of *Bmpr1a* or *Acvr1* leads to imbalance between bone formation and bone resorption resulting in an increase of bone mass. Early studies using 2T3 cell line also suggest that BMPR1A and BMPR1B mediate different signals responsible for osteoblast differentiation^19^. Overexpression of truncated BMPR1A in 2T3 cells enhances bone matrix formation in the absence of BMP-2, whereas overexpression of truncated BMPR1B completely blocks BMP-2-induced mineralized bone matrix formation, and expression of constitutively active BMPR1B induces mineralized bone matrix formation without addition of BMP-2^19^. In the current study, we demonstrated that deletion of *Bmpr1b* showed opposite bone phenotype compared from disruption of *Acvr1* or *Bmpr1a*. However, we observed the osteopenic phenotype only in males, but not in females. One possible explanation is the difference in hormonal regulation between the genders^20^. The other possibility is the difference in adaptation between the genders, e.g. bones in different genders respond differently to exercise^21^,^22^. In addition, the trabecular bone phenotypes we found appeared within a short time window (only at 8-week-old, but not at new born stage or 11-week- old) (Supplementary Figs S1 and S5). These findings suggested that the bone phenotype in *Bmpr1b* null mice is gender- and age-dependent.

Different from BMPR1A and ACVR1, deletion of *Bmpr1b* did not change osteoblast activity or osteoclast activity *in vivo* (Fig. 1). In addition, deletion of *Bmpr1b* did not result in consistent changes of expressions of osteoblast/osteoclast marker genes, or genes related with BMP and WNT signaling in cultured pre-osteoblasts and tibiae (Supplementary Figs S9 and S10). However, deletion of *Bmpr1b* compromised the differentiation ability of bone marrow mesenchymal progenitors towards osteoblasts *in vitro* (Figs 4 and 5). Our results suggested that BMPR1B has distinct functions in bone compared with ACVR1 and BMPR1A. The distinct biological functions may be due to the expression pattern of the three BMP type I receptors. *Bmpr1a* and *Acvr1* are widely expressed in a variety of tissues during development and in multiple adult tissues^23^,^24^. In contrast, the expression of *Bmpr1b* is restricted in early mesenchymal cells and differentiated chondrocytes^23^,^25^. Our results suggested that the relative expression ratios between *Bmpr1b* and the other BMP type I receptors were much higher in bone marrow mesenchymal progenitors compared with those in calvaria (Fig. 6G). This may be the reason why deletion of *Bmpr1b* predominantly influences mesenchymal progenitors, but not calvarial pre-osteoblasts.

It has been reported that there are skeletal stem cells (SSCs) residing in the bone marrows, composing a very small portion of the bone marrow (CD45−, TER119−, Tie2−, alphaV+, Thy−, 6C3−, CD105−, CD200+; alternatively, CD45−, CD31−, Ter119−, CD105+, Grem1+), which are self-renewing, multipotent progenitors for skeletal lineages, which can give rise to cartilage, bone, and stroma^26^,^27^,^28^. SSCs exist in both fetal and postnatal mice, including new born and adult stages^26^. The bone marrow mesenchymal progenitors used in the current study are from flushed bone marrows, thus it is likely that the cells we analyzed in Figs 4, 5, 6 contain SSCs. Since SSCs express BMP ligands and receptors (including *Bmpr1b*), it is a formal possibility that BMPR1B may play a critical role in this population to maintain bone mass. However, little is known how BMP signaling regulates functions of SSCs. It is an interesting and important future endeavor to examine the role of BMP signaling in this specific cell population that may be responsible for the transient bone phenotype in the *Bmpr1b* mutant mice.

We need to be mindful to consider differences in the genetic models when functions of BMPR1B in skeletogenesis are compared with those of other type I BMP receptors. Here, we analyzed bone phenotypes found in conventional mutant mice for *Bmpr1b*, whereas we previously reported bone phenotypes found in osteoblast-specific mutant mice for *Bmpr1a* or *Acvr1*^6^,^7^,^8^,^9^,^10^, since conventional mutations for these genes cause early embryonic lethality^11^,^12^. First, since osteoblast-specific promoters such as 3.2-kb *Col1* and *Og2* were used to drive Cre in these cases, it is unlikely that bone marrow mesenchymal progenitors fully lose BMPR1A or ACVR1 signaling activity. Thus, it is still possible that if appropriate Cre mouse lines were used that direct Cre activity in bone marrow mesenchymal progenitors, conditional KO mice for *Bmpr1a* or *Acvr1* might develop osteopenic phenotypes similar to those found in *Bmpr1b* mutant mice. Our western blot results support this hypothesis; deletion of each of the three BMP type I receptors results in decreased P-SMAD1/5/9 levels, especially in the case of *Bmpr1a* (Fig. 6A–F). Second, in *Bmpr1b* KO mice we analyzed here, all types of cells including osteoblasts and osteoclasts lose BMPR1B signaling. As we reported, BMP signaling in osteoblasts influences osteoclast differentiation *in vivo*^7^. The inconsistency of osteoclast behavior observed from *in vitro* and *in vivo* in our current study may be explained by the facts that *Bmpr1b* KO osteoclasts *in vivo* may receive signals from *Bmpr1b* KO osteoblasts to show different characteristics found in culture. Interestingly, we reported that osteoblastic bone formation activity is increased in the osteoclast-specific KO for *Bmpr1a* mice even though osteoblasts in these mice remain as WT^29^. These data suggest that BMP signaling in osteoclasts somehow influences osteoblast functions. Thus it is possible to speculate that *Bmpr1b* mutant osteoblasts may be influenced by *Bmpr1b* osteoclasts *in vivo*. Nonetheless, we clearly demonstrated that BMP signaling through three different type I BMP receptors show different responses in their SMAD signaling activity in primary calvarial pre-osteoblasts when prompted by BMP ligands in culture (discussed further below). This is an interesting future investigation to uncover molecular mechanisms of how BMPR1B signaling regulates osteoblast-osteoclast communication.

It is surprising that deletion of *Bmpr1b* in calvarial pre-osteoblasts sensitized the cells to BMP stimulation to reach the peak level of P-SMAD1/5/9 at 5 min after BMP stimulation, while the increased P-SMAD1/5/9 did not lead to increased expression of osteoblast differentiation marker genes, despite the fact that increased P-SMAD1/5/9 translocated into nucleus (Fig. 3, Supplementary Figs S9 and S10). One potential explanation is an effect of phosphorylation of a linker region of R-SMADS. It is known that phosphorylation of a linker region of R-SMADS by MAPKs suppresses their translocation to the nucleus and transcriptional activity^30^. Our results showed that the level of P-ERK1/2 in *Bmpr1b* KO cells was lower at early time points upon BMP stimulation. Thus it is possible that decreased level of P-ERK1/2 in KO cells resulted in decreased phosphorylation of linker region of R-SMADS, resulting in higher level of P-SMAD nuclear translocation. Another possible explanation is an involvement of inhibitory SMADS (I-SMADS) or transcriptional repressors in the nucleus. I-SMADS inhibit TGF-β/BMP signaling by interacting with SMAD DNA-binding partners or binding to SMAD-responsive elements directly, thus inhibiting the formation of SMAD-dependent transcription complexes and TGF-β/BMP mediated gene activation^31^,^32^,^33^,^34^. Transcriptional repressing factors *Ski* and *SnoN* disrupt the formation of functional SMAD-DNA complex and thereby inhibit target gene expression^35^. BMPR1B signaling may be involved in some of those mechanisms, however, further investigations are necessary to draw any conclusions.

In summary, our study identified the role of BMPR1B in bone, which is distinct from BMPR1A and ACVR1. We demonstrated that deletion of *Bmpr1b* inhibited early osteoblastic differentiation of bone marrow mesenchymal progenitors, probably being one of the reasons leading to osteopenic phenotype. We also found a distinct role of BMPR1B in transducing BMP signaling in calvarial pre-osteoblasts. This study provides a novel insight into the regulation of bone remodeling.

## Methods

### Mice

Heterozygous *Bmpr1b* null mice (*Bmpr1b*^+/−^)^13^ were bred to generate homozygous mutant mice (*Bmpr1b*^−/−^) on the mixed background (129/SvEv × C57B1/6 J). Both wild type (*Bmpr1b*^+/+^) and mutant (*Bmpr1b*^−/−^) animals within the same littermates were sacrificed at newborn stage, 8 or 11 weeks old. Homozygous floxed mice for *Bmpr1a* or *Acvr1* that were also homozygous for ROSA26 reporter (R26R) were bred with ubiquitin (Ubi) Cre-ER^TM^ transgenic mice (No. 008085, The Jackson Laboratory) that were also heterozygous null for *Bmpr1a* or *Acvr1*^11^,^12^,^36^,^37^,^38^. All animal experiments were performed in accordance with the policy and federal law of judicious use of vertebrate animals as approved by the University Committee on Use and Care of Animals (UCUCA) at University of Michigan.

### Micro-computed tomography (micro-CT)

Femora were embedded in 1% agarose^39^ and placed in a 19 mm diameter tube and scanned over the entire length of the bones using a micro-CT system (μCT100 Scanco Medical, Bassersdorf, Switzerland). Scan settings were: voxel size 10 μm, medium resolution, 70 kVp, 114 μA, 0.5 mm AL filter, and integration time 500 ms. A 0.5 mm region of trabecular compartment was analyzed immediately below the growth plate using a fixed global threshold of 26% (260 on a grayscale of 0–1000, or 569 mg HA/ccm); and a 0.3 mm region of cortical compartment around the midpoint was analyzed using a fixed global threshold of 36% (360 on a grayscale of 0–1000, or 864 mg HA/ccm). Trabecular bone volume fraction (BV/TV), trabecular thickness (Tb. Th), trabecular number (Tb. N), trabecular separation (Tb. Sp), cortical bone volume fraction (BV/TV), and cortical porosity (1-BV/TV) were analyzed using the manufacturer’s evaluation software.

### Histology and histomorphometry

For hematoxylin and eosin (HE) and tartrate-resistant acid phosphatase (TRAP) staining, femora were fixed in 4% paraformaldehyde, decalcified with 10% EDTA, and embedded in paraffin. Longitudinal sections of femora were made at 7 μm, and stained for HE and TRAP.

Static histomorphometry was performed on sections taken from the femora in a blinded, nonbiased manner using Image J (1.49P). Regions of interest (ROIs) were confined to the secondary spongiosa and restricted to a square area (1.1 mm in length and width) 300 μm proximal to the growth plate of the distal femora.

For dynamic histomorphometry, 8-week-old mice were given calcein (0.01 mg/g) in 2% NaHCO_3_ intraperitoneally 7 and 2 days before dissection. Femora were dissected and fixed in 4% paraformaldehyde. The undecalcified femora were embedded in 8% gelatin and 10 μm longitudinal sections were made. The measurements were performed at distal femur trabecular bone.

All static and dynamic parameters were measured according to the Report of the American Society of Bone and Mineral Research Histomorphometry Nomenclature Committee^40^.

### Calvarial pre-osteoblast culture and BMP stimulation

Primary calvarial pre-osteoblasts were isolated from 1- to 3-day-old mice as reported^41^. For osteogenic differentiation, cells were seeded at 2.5 × 10^4^ cells/cm^2^ and allowed for adhesion in α-MEM containing 10% fetal bovine serum (FBS, Denville) overnight. On the second day, the medium was changed to differentiation medium (DM) (α-MEM containing 10% FBS, 10 mM β-glycerophosphate (Sigma-Aldrich) and 50 μg/ml L-ascorbic acid (Sigma-Aldrich)) either with or without 100 ng/ml recombinant human bone morphogenetic protein 2 (rhBMP-2, R&D Systems) for the indicated days and stained for alkaline phosphatase and alizarin red.

To stimulate BMP signaling, cells were starved (α-MEM containing 0.5% FBS) overnight before treatment with 100 ng/ml rhBMP-2 for the indicated times. Then the cells were fixed for immunocytochemistry or lysed for protein harvest.

### Isolation and culture of bone marrow mesenchymal progenitors

Bone marrow mesenchymal progenitors were isolated from femora and tibiae of 8-week-old mice as reported^41^. Differentiation and BMP stimulation of bone marrow mesenchymal progenitors was performed as mentioned above.

### Tamoxifen treatment and cell sorting

Calvarial pre-osteoblasts and bone marrow mesenchymal progenitors were harvested from *Bmpr1a* or *Acvr1* fx/+ :Ubi Cre-ER^TM^ (+)/(−):*R26R*/+ and fx/−:Ubi Cre-ER^TM^ (+)/(−):*R26R*/+ mice. To disrupt *Bmpr1a* or *Acvr1* in culture, Ubi Cre-ER^TM^ activity was induced by administering 100 ng/ml of (Z)-4-hydroxytamoxifen (TM) (Sigma-Aldrich) for 6 days^8^. To isolate Cre-dependent recombined populations, cells were then stained with fluorescein di β-D-galactopyranoside (FDG) (Invitrogen). The cells with the *Acvr1* or *Bmpr1a* gene recombined by Cre activity became positive for fluorescence (514 nm), while those without recombination were negative. The cells were sorted for fluorescence positive populations using a FACSAria III cell sorter. Fx/+ fluorescence positive populations were used as controls, and fx/- fluorescence positive populations were used as mutants.

### Isolation and culture of osteoclasts

Bone marrow cells were obtained by flushing from femora of 8-week-old mice^42^. After cultured overnight in α-MEM containing 10% FBS (Denville), non-adherent cells were harvested as bone marrow mononuclear cells (BMMCs). BMMCs were cultured with 30 ng/ml Recombinant Murine Macrophage Colony Stimulating Factor (rmM-CSF) (PEPROTECH) to proliferate, and cells at this stage were used as osteoclast precursors. To generate mature osteoclasts, osteoclast precursors were cultured with 30 ng/ml M-CSF and 50 ng/ml Recombinant Murine soluble Receptor Activator of NF-κB Ligand (rmRANKL) (PEPROTECH)^43^. Medium was changed every other day.

To determine the proliferation of osteoclast precursors, BMMCs were cultured with 30 ng/ml M-CSF. After 6 days of culture, the number of osteoclast precursors was counted. To determine the differentiation of osteoclasts, 30 ng/ml M-CSF and gradient concentrations of RANKL (10–50 ng/ml) were used. After 6 days of differentiation, TRAP staining was performed, and the number of osteoclasts as well as the number of nuclei per osteoclasts were counted. To determine the survival of osteoclasts, after 6 days of differentiation using 30 ng/ml M-CSF and 50 ng/ml RANKL, the medium was removed and the cells were washed twice with α-MEM. Then the cells were continued culturing in α-MEM without M-CSF and RANKL for the indicated hours, followed by TRAP staining. The number of osteoclasts remained in the culture was counted. To determine the bone resorption function of osteoclasts, osteoclasts precursors were seeded onto Osteo Assay Surface (Corning) and underwent differentiation using 30 ng/ml M-CSF and 50 ng/ml RANKL. After 6 days of culture, the resorption area was measured and was normalized by osteoclast number.

### Histochemistry staining of cell cultures

After bone marrow mesenchymal progenitors or calvarial pre-osteoblasts were cultured for 7 and 14 days, cells were stained for alkaline phosphatase (ALP) using alkaline phosphatase kits (Sigma-Aldrich) according to manufacturer’s instructions. Mean density of the staining was measured using Image J. After culturing for 21 days, cells were stained with 40 mM alizarin red S (Sigma-Aldrich) for 10 min at room temperature. Ten % w/v cetylpyridinium chloride (Sigma-Aldrich) was used to dissolve bound alizarin red S and optical density was measured at 562 nm. Osteoclasts were stained for TRAP using acid phosphatase kit (Sigma-Aldrich) according to manufacturer’s instructions. Osteoclasts were defined as TRAP positive cells with three or more nuclei.

### Immunocytochemistry staining

After BMP stimulation, cells were fixed in 4% PFA at room temperature for 15 min, permeabilized using 100% methanol at −20 °C for 10 min. The cells were incubated with rabbit anti-pSMAD1/5/9 (1:800, Cat. No. 13820, Cell Signaling Technology), rabbit anti-Smad1/5/9 (1:100, sc-6031-R, Santa Cruz Biotechnology) at 4 °C overnight. Alexa Fluor^®^ 594 donkey anti-rabbit IgG (H + L) antibody (1:200, Invitrogen) were used as secondary antibody. The staining was mounted with ProLong^®^ Gold antifade reagent with DAPI (Invitrogen). The fluorescent images were taken by Olympus DP70 camera with the same exposure time in the same experiment. The mean density in the nucleus or cytoplasm was measured using Image J.

### Immunoblot analysis

Cells were lysed in RIPA buffer (20 mM Tris-HCl, 0.1% SDS, 1% Triton X-100, 1% sodium deoxycholate) containing protease inhibitor (Roche). The resulting lysates were run on 4–15% or 10% Mini-PROTEAN^®^ TGX^TM^ Gel (Bio-Rad) and transferred to Amersham Hybond-P membrane (GE Healthcare). The following antibodies were used (all are from Cell Signaling Technology): rabbit anti-pSMAD1/5/9 (1:1000, Cat. No. 13820), rabbit anti-phospho-p44/42 MAPK (pERK1/2) (1:1000, Cat. No. 4376), rabbit anti-p44/42 MAPK (ERK1/2) (1:1000, Cat. No. 4695), rabbit anti-phospho-p38 MAPK (1:1000, Cat. No. 4631), rabbit anti-p38 MAPK (1:1000, Cat. No. 9212), and rabbit anti-GAPDH (1:2000, Cat. No. 2118). SuperSignal West Pico Chemiluminescent substrate (Thermo Scientific) was used to detect bound antibodies.

### RNA isolation and quantitative real-time RT-PCR

Total mRNA was isolated from tibiae, cultured calvarial pre-osteoblasts or bone marrow mesenchymal progenitors using TRIzol reagent (Life Technologies). For quantitative real-time RT-PCR analyses, equal amounts of RNA were reverse-transcribed using Superscript III first-strand synthesis System (Invitrogen) with oligo (dT) as a primer. The resulting cDNA templates were subjected to quantitative PCR using TaqMan probes (*Gapdh*: Mm99999915_g1; *Runx2*: Mm00501578_m1; *Sp7*: Mm00504574_m1; *Alpl*: Mm00475831_m1; *Bglap*: Mm01741771_g1; *Ibsp*: Mm00492555_m1; *Dmp1*: Mm00803833_g1; *Col1a2*: Mm00483888_m1; *Bmpr1b*: Mm00432117_m1; *Bmpr1a*: Mm00477650_m1; *Acvr1*: Mm01331069_m1; *Ctnnb1*: Mm00483039_m1; *Lrp5*: Mm01227476_m1; *Axin2*: Mm00443610_m1; *Lef1*: Mm00550265_m1; *Dkk1*: Mm00438422_m1; *Dkk2*: Mm00445025_m1; *Sost*: Mm00470479_m1; *Tnfsf11*: Mm00441908_m1; *Tnfrsf11b*: Mm00435452_m1; *Id1*: Mm00775963_g1; *Plod1*: Mm01255760_m1; *Plod2*: Mm00478767_m1; *Plod3*: Mm00478798_m1; *Lox*: Mm00495386_m1; *Loxl1*: Mm01145738_m1; *Loxl2*: Mm00804740_m1; *Loxl3*: Mm01184865_m1; *Loxl4*: Mm00446385_m1; *Mmp9*: Mm00442991_m1; *Acp5*: Mm00475698_m1; *Ctsk*: Mm00484039_m1) by ABI PRISM 7500 (Applied Biosystems). Data were normalized to *Gapdh* expression using the 2^−ΔΔCt^ method.

### Statistical analysis

Data were expressed as means ± standard deviation of triplicate measurements with all experiments independently repeated at least three times. Unpaired Student’s two-tailed t-tests were used to evaluate statistical differences. Values of p < 0.05 were considered significant.

## Additional Information

**How to cite this article**: Shi, C. *et al.* Deletion of BMP receptor type IB decreased bone mass in association with compromised osteoblastic differentiation of bone marrow mesenchymal progenitors. *Sci. Rep.* **6**, 24256; doi: 10.1038/srep24256 (2016).

## Supplementary Material

Supplementary Information
