# Supplementary material for: Deletion of BMP receptor type IB decreased bone mass in association with compromised osteoblastic differentiation of bone marrow mesenchymal progenitors
Source: Sci Rep. 2016 Apr 6;6:24256. doi: 10.1038/srep24256 (PMC4822175; doi:10.1038/srep24256)
Supplement: Supplementary Information [file srep24256-s1.pdf]

Deletion of BMP receptor type IB decreased bone mass in association with compromised osteoblastic differentiation of bone marrow mesenchymal progenitors

Ce Shi [1,2], Ayaka Iura [2], Masahiko Terajima [3], Fei Liu [2], Karen Lyons [4, 5, 6], Haichun Pan [2], Honghao Zhang [2], Mitsuo Yamauchi [3], Yuji Mishina [2, *], Hongchen Sun [1, *]

1 Department of Oral Pathology, School and Hospital of Stomatology, Jilin University, Changchun, 130021, China

2 Department of Biologic and Materials Sciences, University of Michigan, School of Dentistry, Ann Arbor, MI 48109-1078, USA

3 School of Dentistry, University of North Carolina, Chapel Hill, NC 27514, USA

4 Department of Molecular, Cell and Developmental Biology, University of California, Los Angeles, Los Angeles, CA 90095, USA

5 Department of Orthopaedic Surgery, University of California, Los Angeles, CA 90095, USA

6 Orthopaedic Institute for Children, Los Angeles, CA 90007, USA

* Corresponding author: mishina@umich.edu; hcsun@mail.jlu.edu.cn

Supplementary Methods

Serum analyses

Whole blood was harvested from aorta abdominalis, using heparin (30 mg/ml) as anticoagulant. Blood samples were put on ice for 1 h followed by centrifugation at 4500 rpm for 30 min at 4 ℃. Supernatants were harvested as serum and were stored at -80 ℃ for further biochemistry analyses. Serum levels of N-terminal propeptide of type 1 procollagen (P1NP) (IDS), collagen type 1 cross-linked C-telopeptide (CTX-1) (IDS), calcium and phosphate were measured.

Amino acid and collagen cross-link analyses

Tibiae (excluding bone marrow) or cultured calvarial pre-osteoblasts were harvested and pulverized to fine powder under liquid N2 using a Spex Freezer Mill (Spex Metuchen). The sample was subjected to amino acid analysis on a Varian high performance liquid chromatography system (Prostar 240/310, Varian, Walnut Creek)[1]. Hydroxyproline (Hyp) was measured, and the collagen composition was calculated and expressed as a percentage of total protein. The extent of lysine hydroxylation (Hyl) of collagen was calculated as the Hyl residues per 300 residues of Hyp and expressed as moles/mole of collagen (mol/mol of collagen)[2].

To analyze collagen cross-link, the bone samples were demineralized with 0.5 M EDTA and 0.05 M Tris-HCl (pH 7.4) for 2 weeks, reduced with standardized NaB^3H_4 [3], and hydrolyzed with 6N HCl. The culture samples were washed with cold phosphate buffered saline and distilled water, lyophilized, reduced and hydrolyzed in the same manner. The hydrolysates with known amounts of Hyp were analyzed

for cross-links on a Varian HPLC system linked to an on-line fluorescence flow monitor (FP-1520, Jasco) and a liquid scintillation flow monitor (500TR series, Packard Instrument, Meriden). The cross-link precursor aldehydes and the major divalent reducible cross-links were analyzed as their reduced forms, i.e. dihydroxynorleucine (DHNL), dihydroxylysinonorleucine (DHLNL) and hydroxylysinonorleucine (HLNL), respectively. The non-reducible cross-links, pyridinoline (Pyr) and deoxypyridinoline (d-Pyr), were also analyzed, as previously reported[4]. All cross-links were quantified as mol/mol of collagen.

Supplementary References

1 Yamauchi, M. & Shiiba, M. Lysine hydroxylation and cross-linking of collagen. *Methods in molecular biology* **446**, 95-108, doi:10.1007/978-1-60327-084-7_7 (2008).

2 Katafuchi, M., Matsuura, T., Atsawasuwan, P., Sato, H. & Yamauchi, M. Biochemical characterization of collagen in alveolar mucosa and attached gingiva of pig. *Connective tissue research* **48**, 85-92, doi:10.1080/03008200601143508 (2007).

3 Yamauchi, M., Katz, E. P. & Mechanic, G. L. Intermolecular cross-linking and stereospecific molecular packing in type I collagen fibrils of the periodontal ligament. *Biochemistry* **25**, 4907-4913 (1986).

4 Sricholpech, M. *et al.* Lysyl hydroxylase 3-mediated glucosylation in type I collagen: molecular loci and biological significance. *The Journal of biological chemistry* **287**, 22998-23009, doi:10.1074/jbc.M112.343954 (2012).

Supplementary Tables

Supplementary Table S1. *Bmpr1b* homozygous mutant mice and controls were born with a normal Mendelian ratio.

	male	female	total number
WT	30	30	60
HET	64	70	134
KO	30	23	53
total number	124	123	247

Supplementary Figure Legends

Supplementary Figure S1. The bone phenotype is not observed at new born stage. Representative histological images of H&E stained sections from new born male and female distal femora.

Supplementary Figure S2. Eight-week-old KO males, but not females, had a decreased body weight. For each group, n=10. * $p<0.05$. WT: wild type; KO: knockout.

Supplementary Figure S3. Other parameters determined by micro-CT and histomorphometry for the femurs from 8-week-old male mice: (A-B) Trabecular parameters: (A) bone mineral density (BMD); (B) structure model index (SMI). (C-E) Cortical parameters: (C) BV/TV; (D) BMD; (E) cortical area. (F-H) Static histomorphometry for the trabecular compartment of distal femurs of 8-week-old male mice: (F) trabecular bone separation (Tb. Sp); (G) osteoblast surface per bone surface (Ob. S/BS); (H) osteoclast surface per bone surface (Oc. S/BS). * $p<0.05$; ** $p<0.01$. WT: wild type; KO: knockout.

Supplementary Figure S4. *Bmpr1b* deletion did not influence bone phenotype in female mice. (A-E) Trabecular parameters were determined by micro-CT for the femurs from 8-week-old female mice: (A) BV/TV; (B) trabecular thickness (Tb.Th) and trabecular separation (Tb. Sp); (C) trabecular number (Tb. N); (D) structure model index (SMI); (E) bone mineral density (BMD) and tissue mineral density (TMD); (F-L) Cortical parameters were determined by micro-CT for the femurs from 8-week-old female mice:

(F) BV/TV; (G) cortical porosity; (H) BMD and TMD; (I) cortical area; (J) cortical thickness; (K) inner cortical perimeter; (L) outer cortical perimeter. For WT, n=3; for KO, n=4. * $p<0.05$. WT: wild type; KO: knockout.

Supplementary Figure S5. The bone phenotype is not observed in 11-week-old male. (A-F) Trabecular parameters were determined by micro-CT for the femora from 11-week-old male mice: (A) trabecular bone volume fraction (bone volume/tissue volume, BV/TV); (B) trabecular number (Tb. N); (C) trabecular thickness (Tb.Th); (D) trabecular separation (Tb. Sp); (E) bone mineral density (BMD); (F) tissue mineral density (TMD); (G-N) Cortical parameters: (G) cortical bone volume fraction (bone volume/tissue volume, BV/TV); (H) cortical porosity (1-BV/TV)%; (I) inner perimeter; (J) outer perimeter; (K) cortical thickness; (L) cortical area; (M) BMD; (N) TMD. (O) Representative H&E stained sections; (P-X) Static histomorphometry for the trabecular compartment of distal femora of 11-week-old male mice: (P) trabecular bone area/tissue area (BA/TA); (Q) trabecular bone number (Tb. N); (R) trabecular bone thickness (Tb. Th); (S) trabecular separation (Tb. Sp); (T) osteoblast number per bone surface (N. Ob/BS); (U) osteoblast surface per bone surface (Ob. S/BS); (V) osteoclast number per bone surface (N. Oc/BS); (W) osteoclast surface per bone surface (Oc. S/BS); (X) eroded surface per bone surface (ES/BS). For WT, n=3; for KO, n=5. * $p<0.05$. WT: wild type; KO: knockout.

Supplementary Figure S6. Biochemical markers from serum and culture medium for bone formation and bone resorption were not changed. (A) serum P1NP level; (B) serum CTX-

1 level; (C) serum calcium and phosphorus levels; (D-E) P1NP levels from culture media of calvarial pre-osteoblasts stimulated without (D) or with (E) BMP-2. WT: wild type; KO: knockout.

Supplementary Figure S7. Osteoblastic differentiation of calvarial pre-osteoblasts was not changed. Calvarial pre-osteoblasts were isolated from newborn mice and subjected to osteoblast differentiation. Cells were treated with or without BMP-2. (A) Alkaline phosphatase (ALP) staining and mean density of the staining after 7 days of differentiation. (B) ALP staining and mean density of the staining after 14 days of differentiation. (C) Alizarin red staining and quantification of the staining after 21 days of differentiation. WT: wild type; KO: knockout.

Supplementary Figure S8. Deletion of *Bmpr1b* did not change the expressions of other BMP type I receptors. Total mRNA was isolated from cultured calvarial pre-osteoblasts treated with or without BMP-2, and quantitative RT-PCR was performed. Expression of *Bmpr1b* (A), *Bmpr1a* (B), and *Acvr1* (C) was calculated. WT: wild type; KO: knockout.

Supplementary Figure S9. Gene expression levels were not changed in cultured calvarial pre-osteoblasts *in vitro*. Calvarial pre-osteoblasts were harvested from 3-day-old mice. Cells were treated with or without 50 ng/ml rhBMP-2. Total mRNA was isolated, and quantitative RT-PCR was performed. For WT, n=3; for KO, n=4. * $p<0.05$. WT: wild type; KO: knockout.

Supplementary Figure S10. Gene expression levels were not changed in tissues from tibiae *in vivo*. Total mRNA was isolated from whole tibiae of both male and female animals at 8 weeks old, and quantitative RT-PCR was performed. For WT, n=3 from both male and female; for KO, n=5 from male and n=3 from female. * $p<0.05$. WT: wild type; KO: knockout.

Supplementary Figure S11. Deletion of *Bmpr1b* did not change the collagen composition or collagen cross-links. Collagen composition (A and D), hydroxylysine (B and E) and collagen cross-links (C and F) were measured by a Varian HPLC system. Samples are from *in vivo* tibiae (A, B and C) and cultured calvarial pre-osteoblasts (D, E and F). WT: wild type; KO: knockout.

Supplementary Figure S12. Gene expressions of the collagen modifying enzymes were not changed. Samples were from male and female tibiae (A-H) or cultured calvarial pre-osteoblasts in the absence or presence of BMP (I-J). * $p<0.05$. WT: wild type; KO: knockout.

Supplementary Figure S13. The uncropped films of Western Blot results in Fig. 3, Fig. 5 and Fig. 6. For all the experiments, detections of P-Smad1/5/9 and GAPDH were performed on the same membrane. The membrane was firstly detected for P-Smad1/5/9, and then probed for GAPDH. The exposure times for P-Smad1/5/9 and GAPDH were different. Detections of P-ERK1/2, ERK1/2, P-P38 and P38 were performed on different

membranes, due to the similar molecular weight. The samples were derived from the same experiment and the gels/blots were processed in parallel.

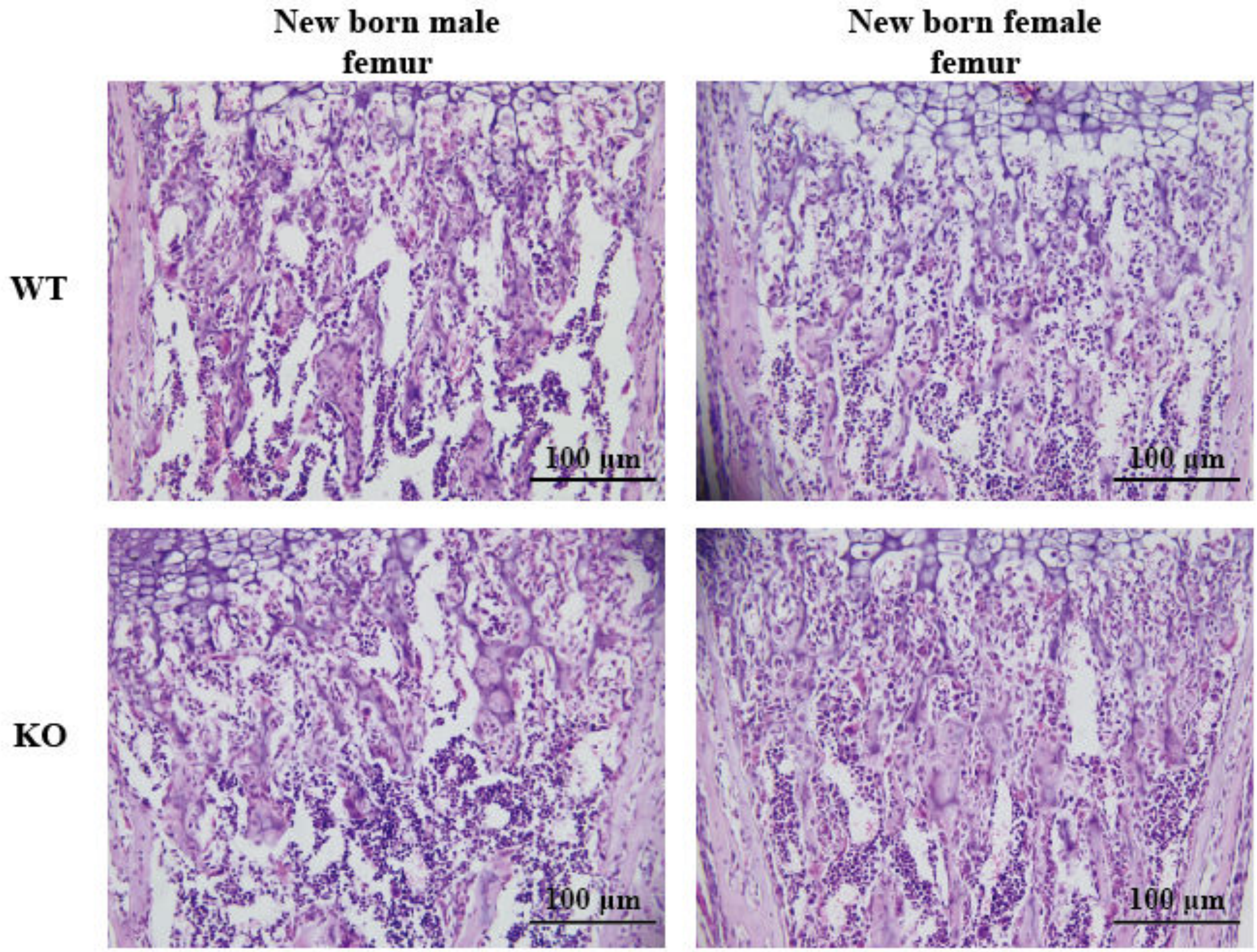

Shi, *et al.*, Supplemental Figure S1

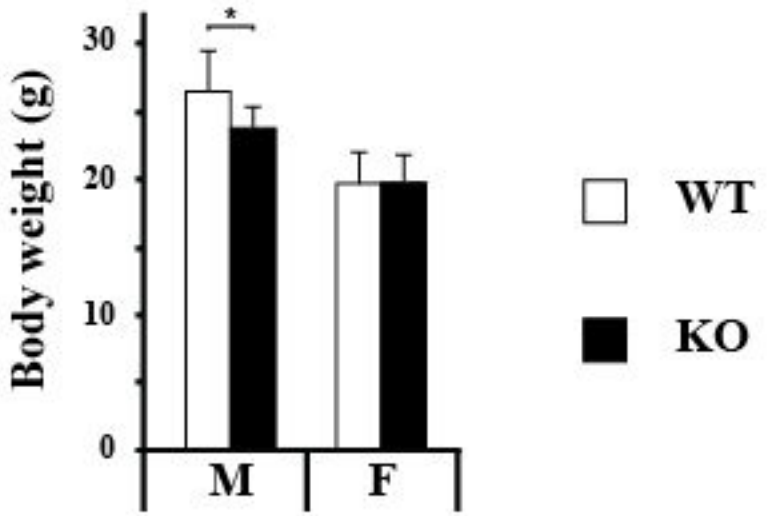

Shi, *et al.*, Supplementary Figure S2

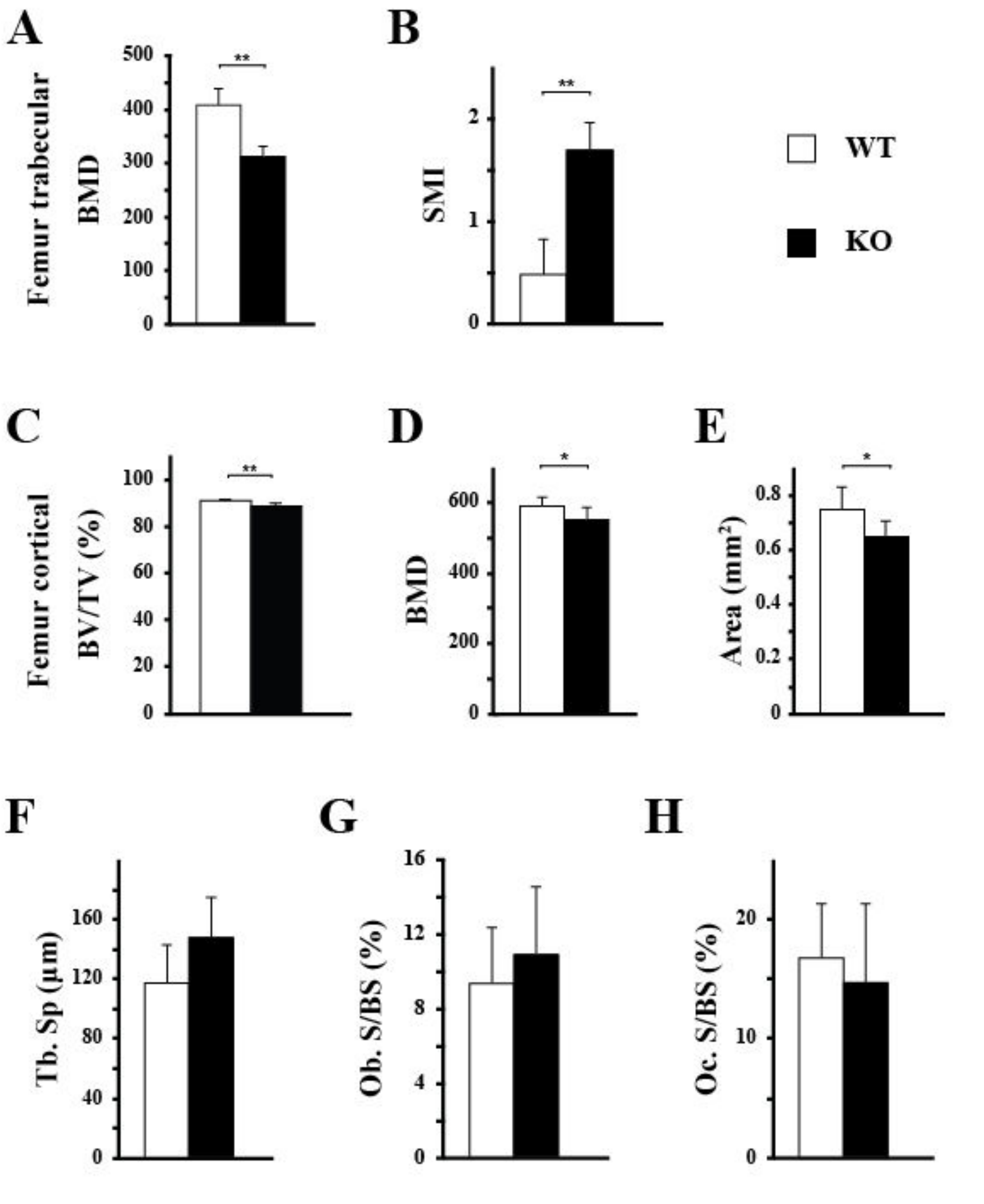

Shi, *et al.*, Supplementary Figure S3

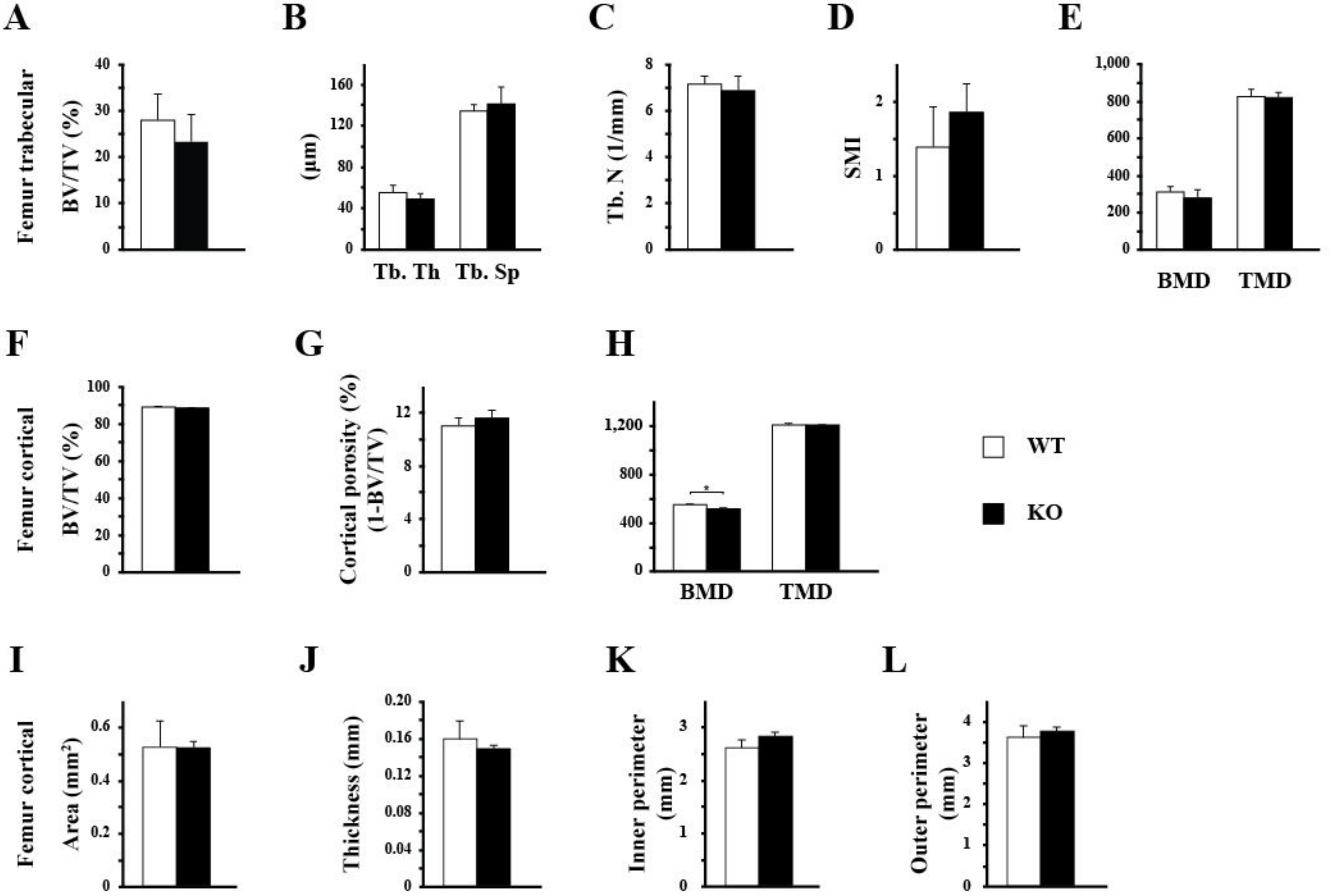

Shi, *et al.*, Supplementary Figure S4

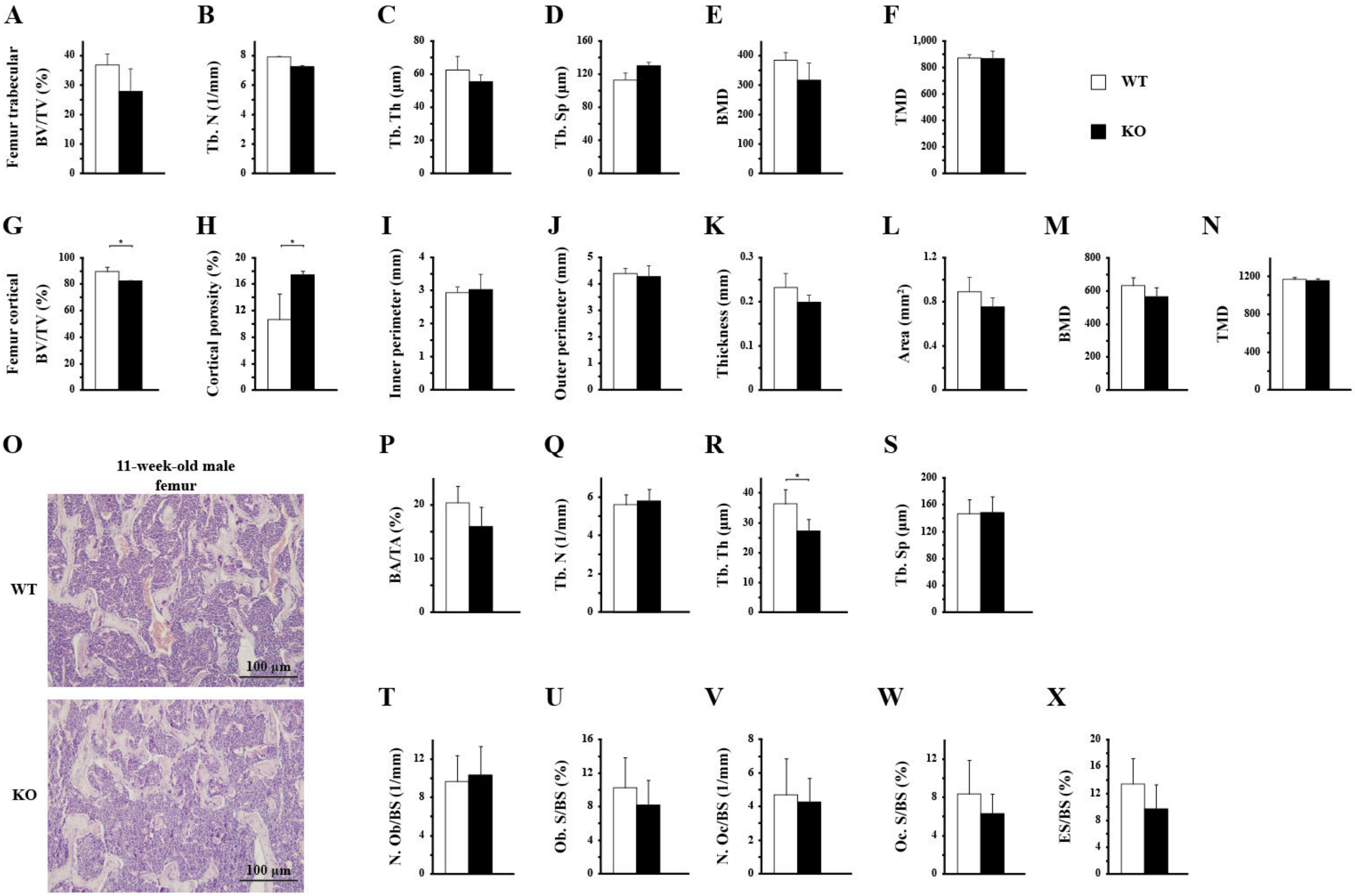

Shi, *et al.*, Supplemental Figure S5

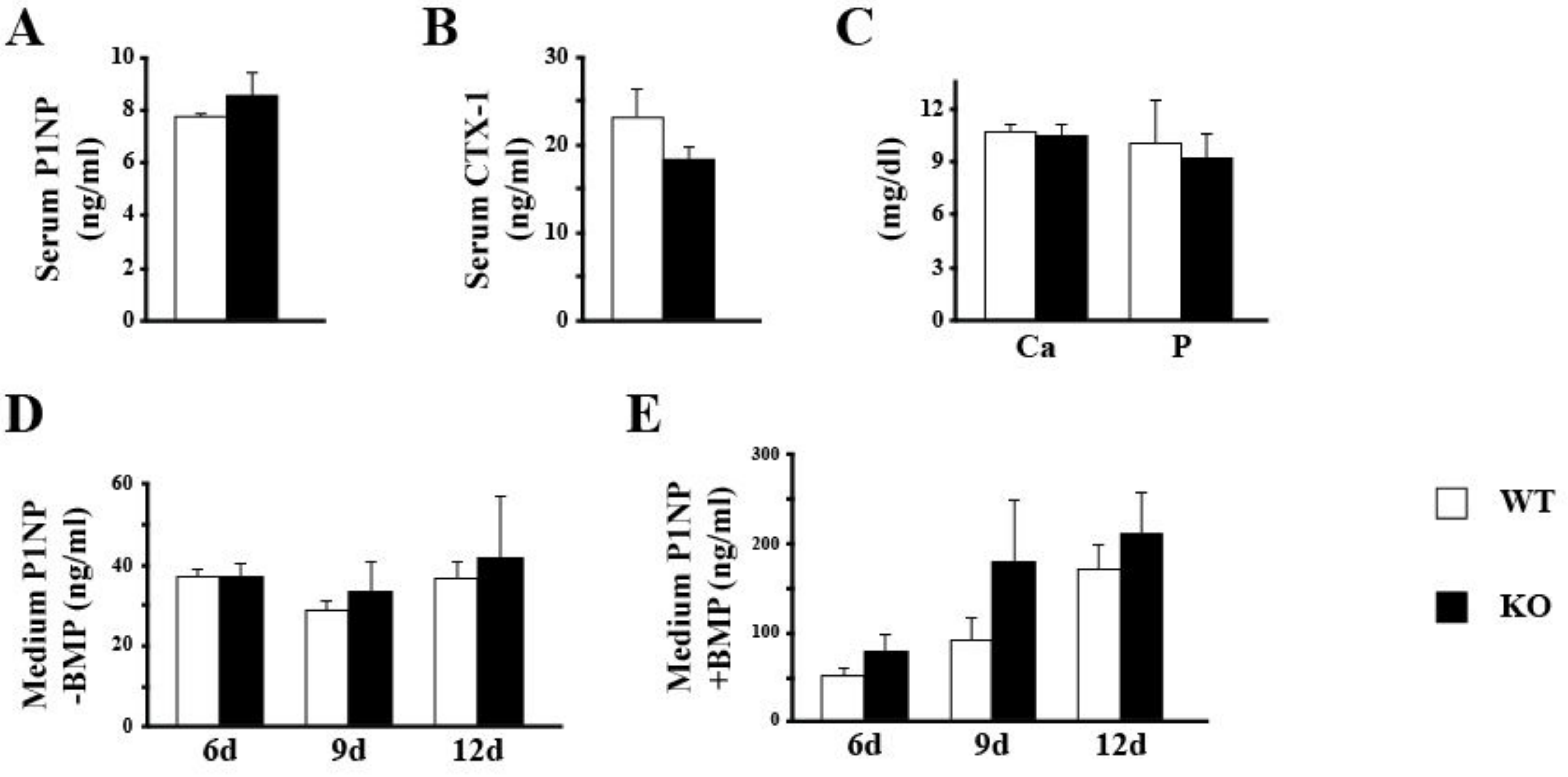

Shi, *et al.*, Supplementary Figure S6

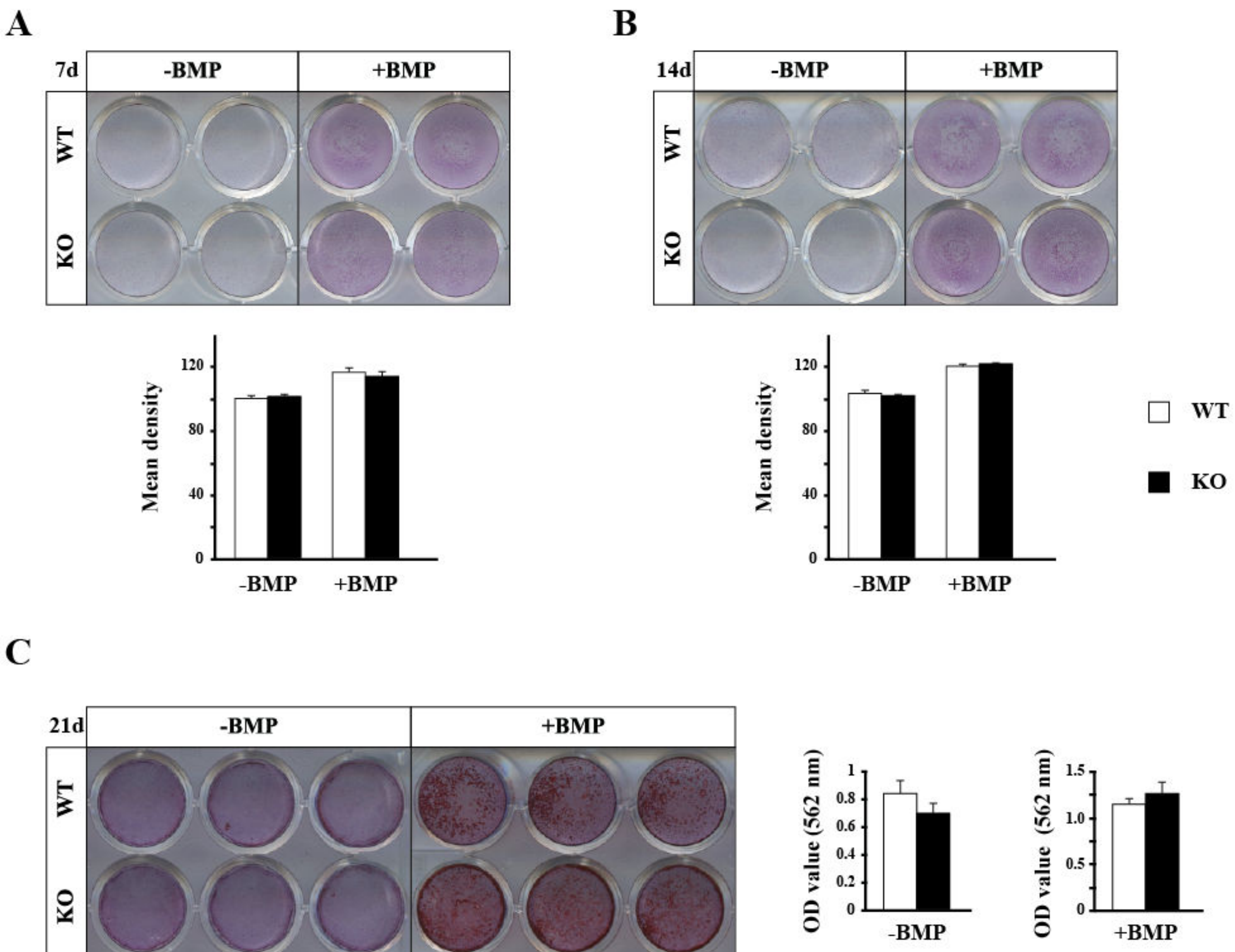

Shi, *et al.*, Supplemental Figure S7

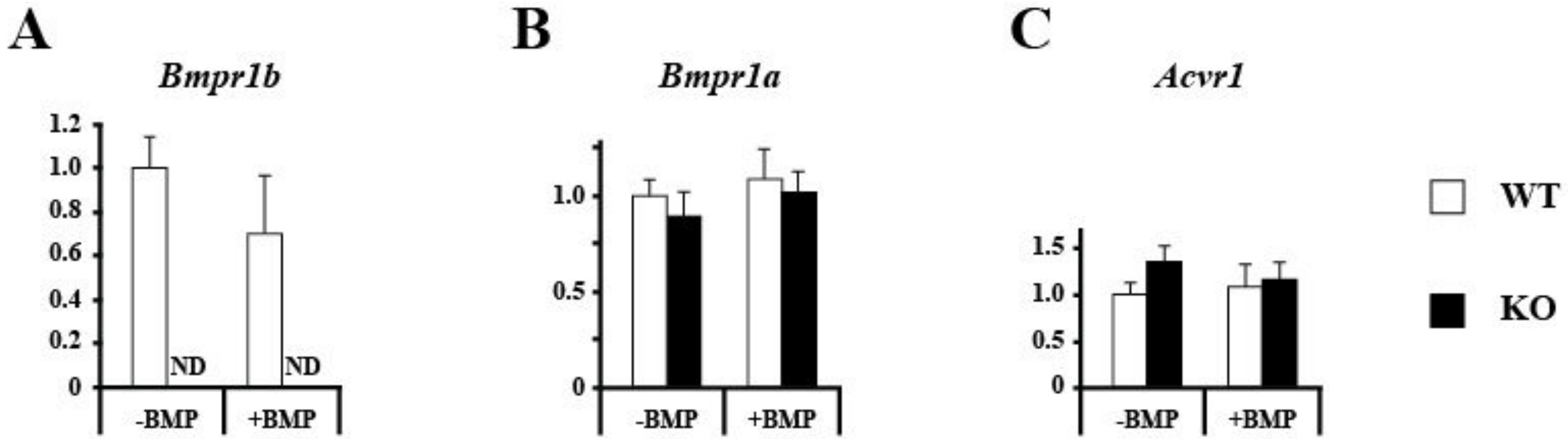

Shi, *et al.*, Supplementary Figure S8

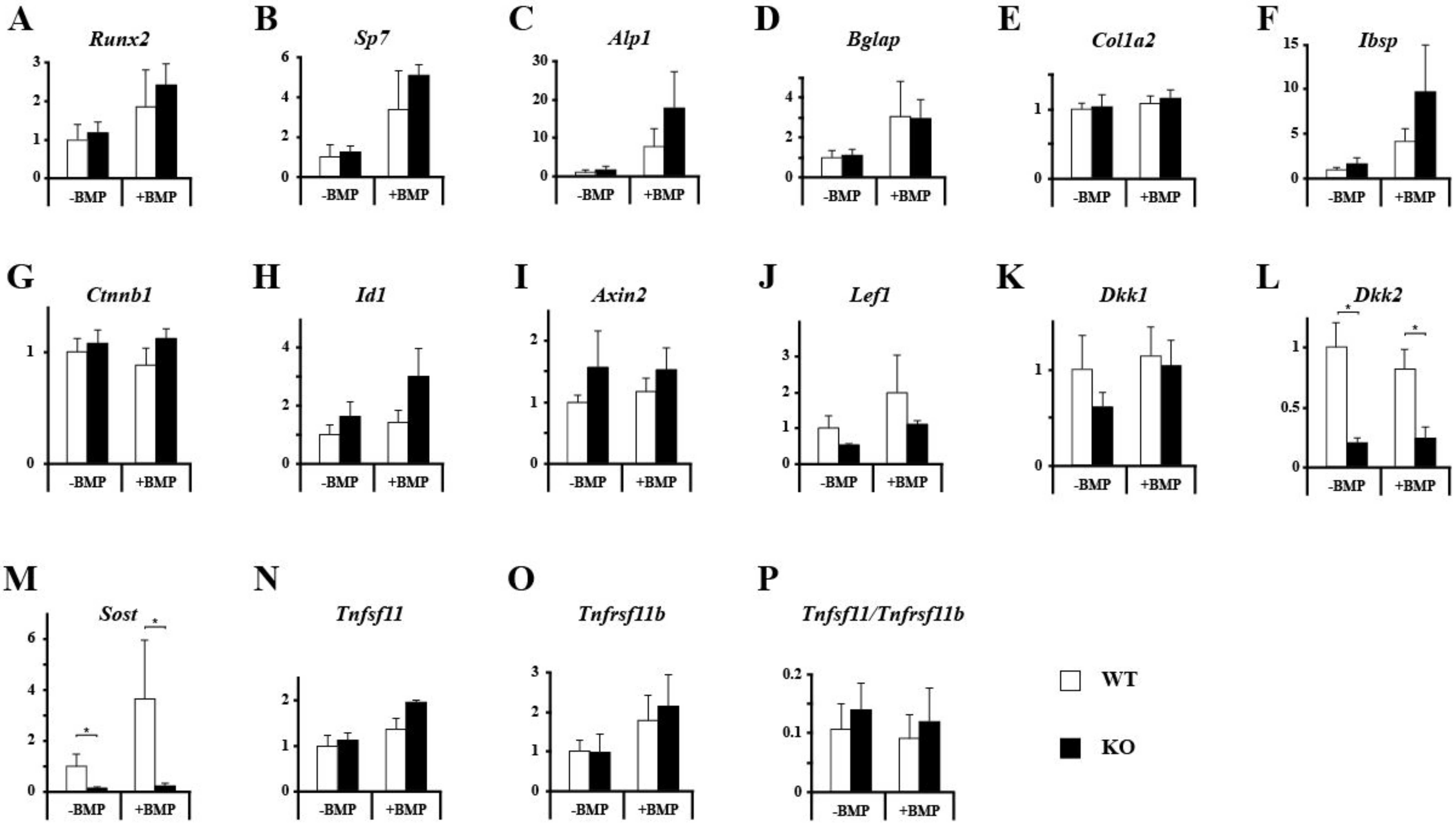

Shi, *et al.*, Supplementary Figure S9

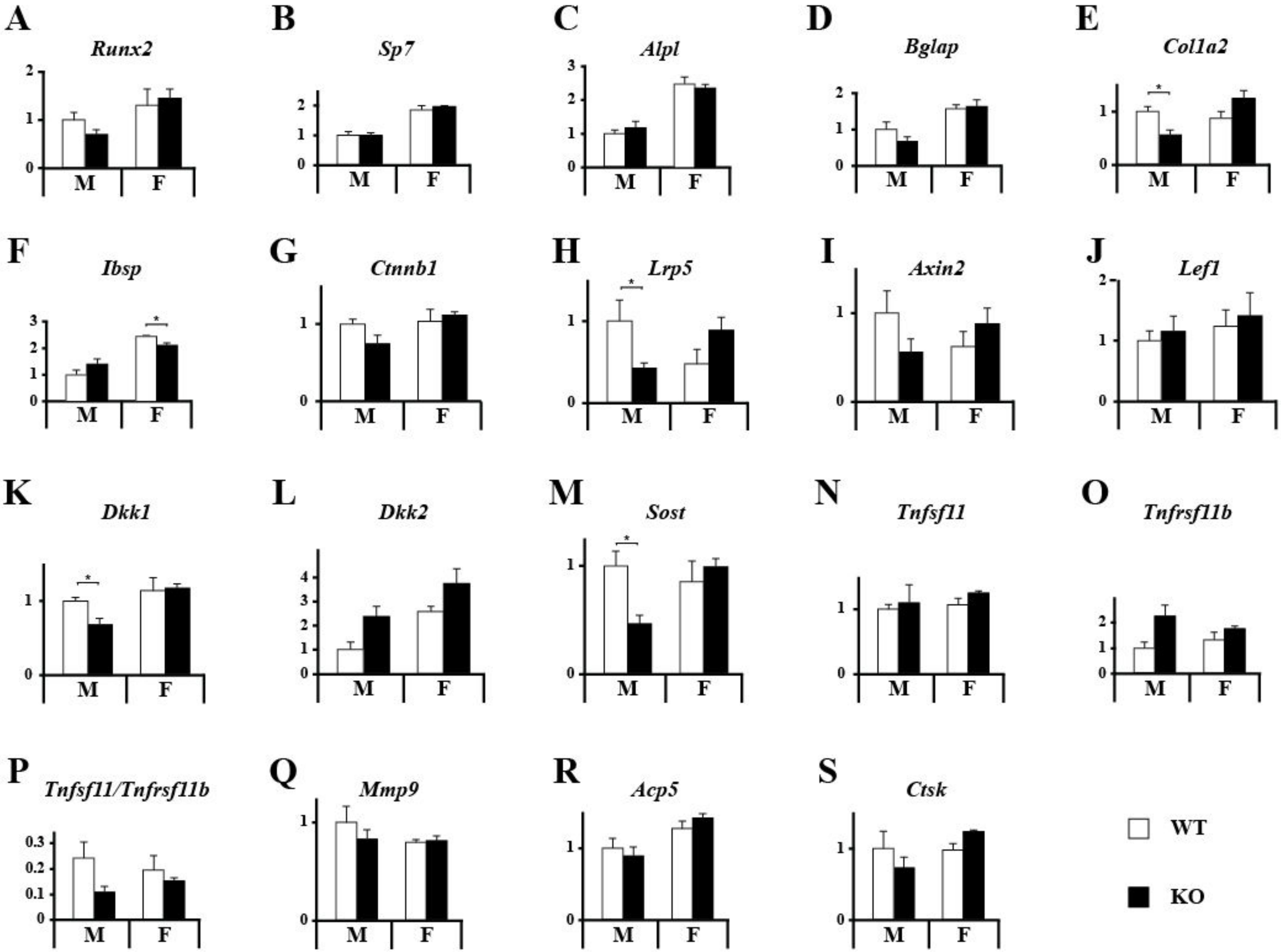

Shi, *et al.*, Supplementary Figure S10

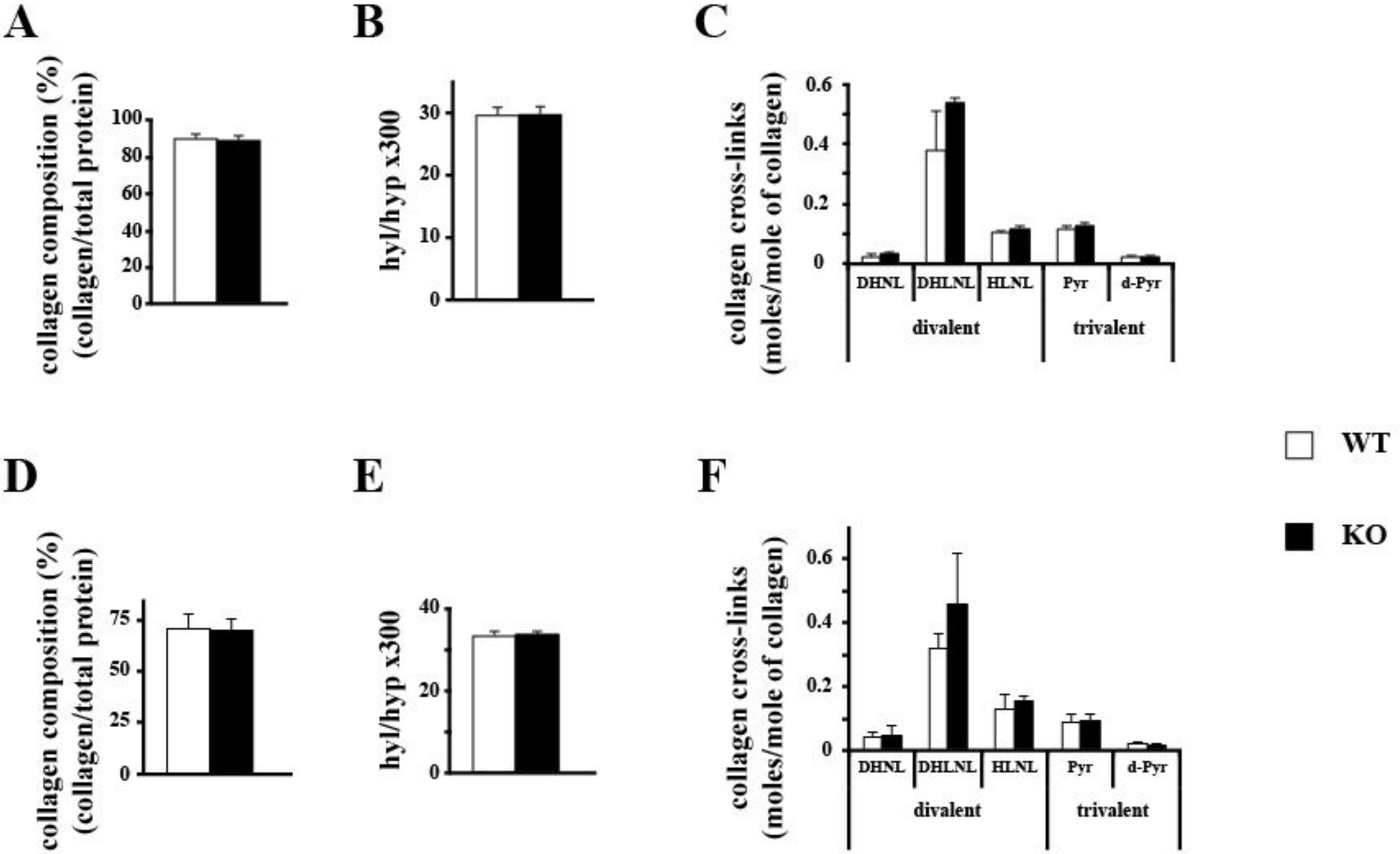

Shi, *et al.*, Supplementary Figure S11

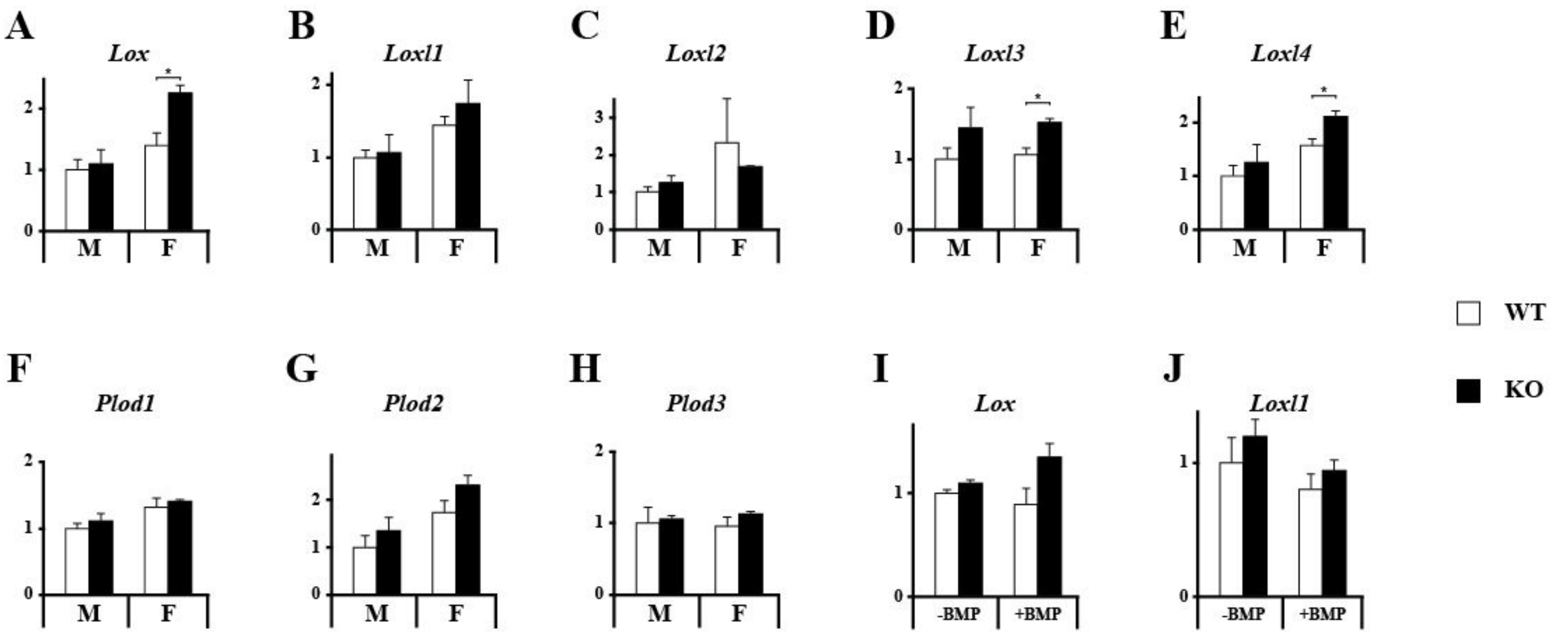

Shi, *et al.*, Supplementary Figure S12

Fig. 3A

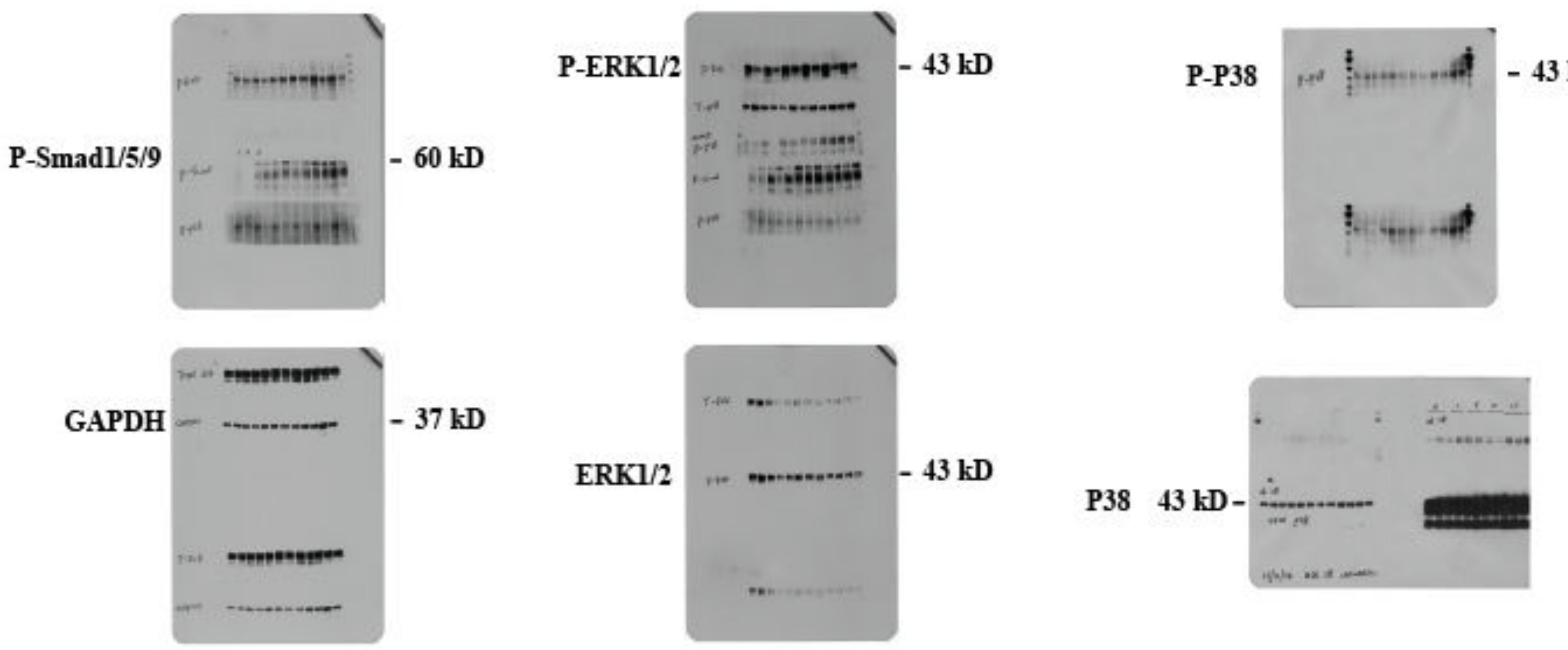

Fig. 3C

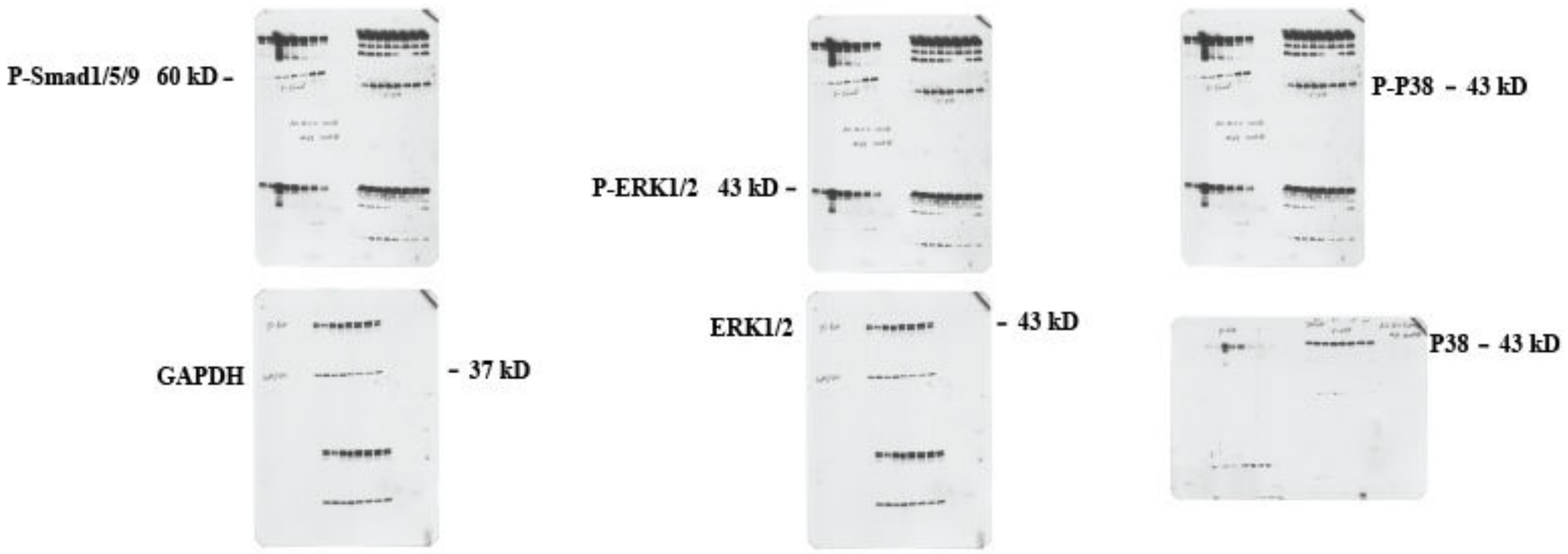

Fig. 3D

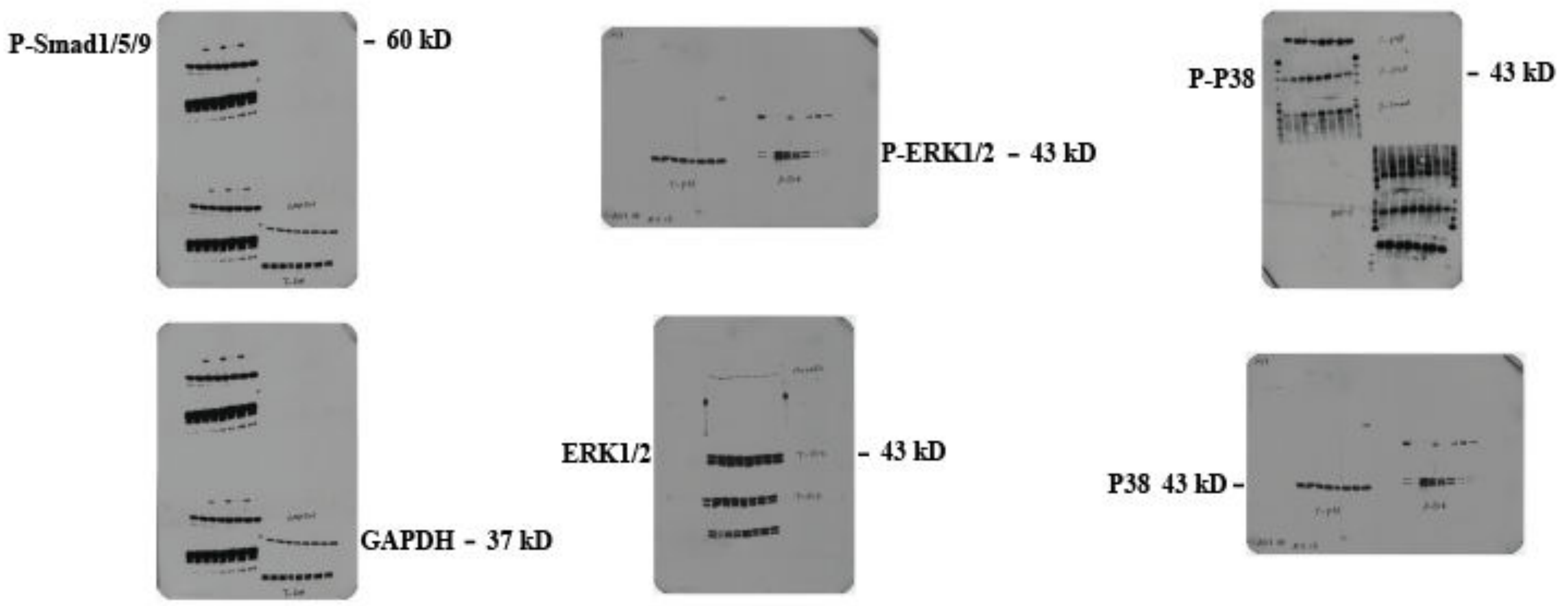

Fig. 6A

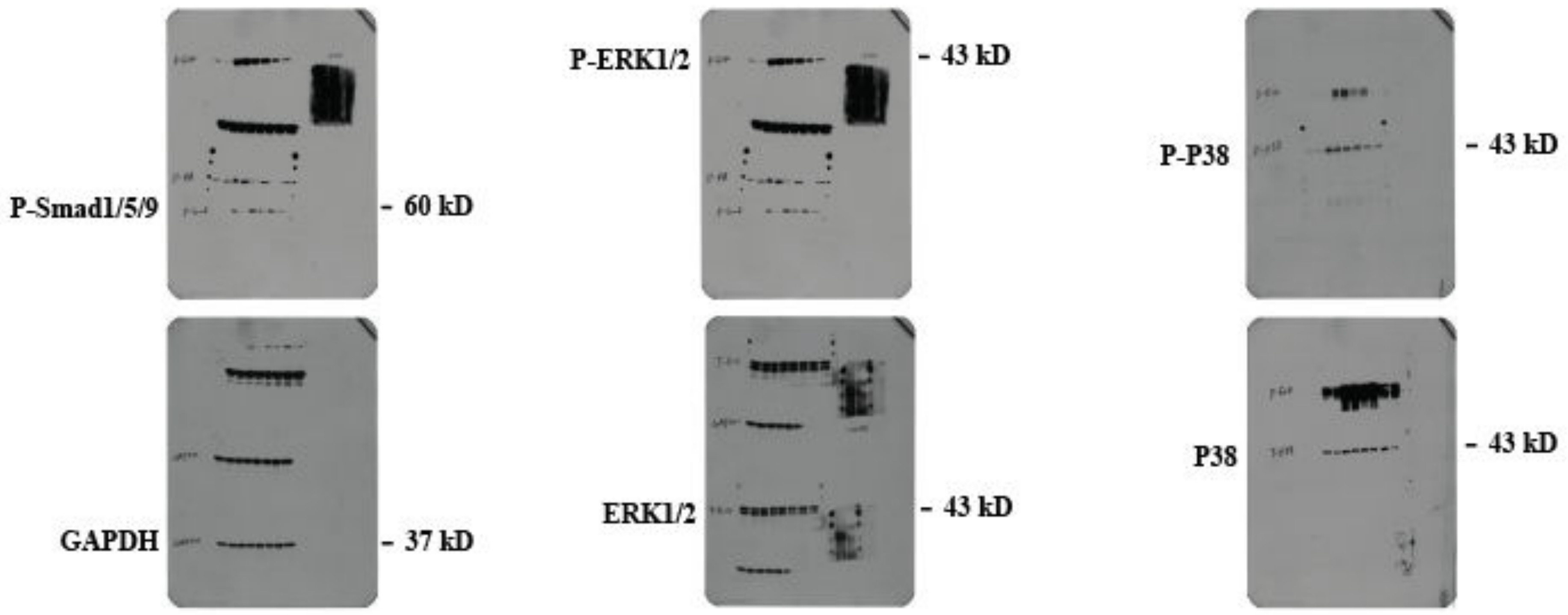

Fig. 6C

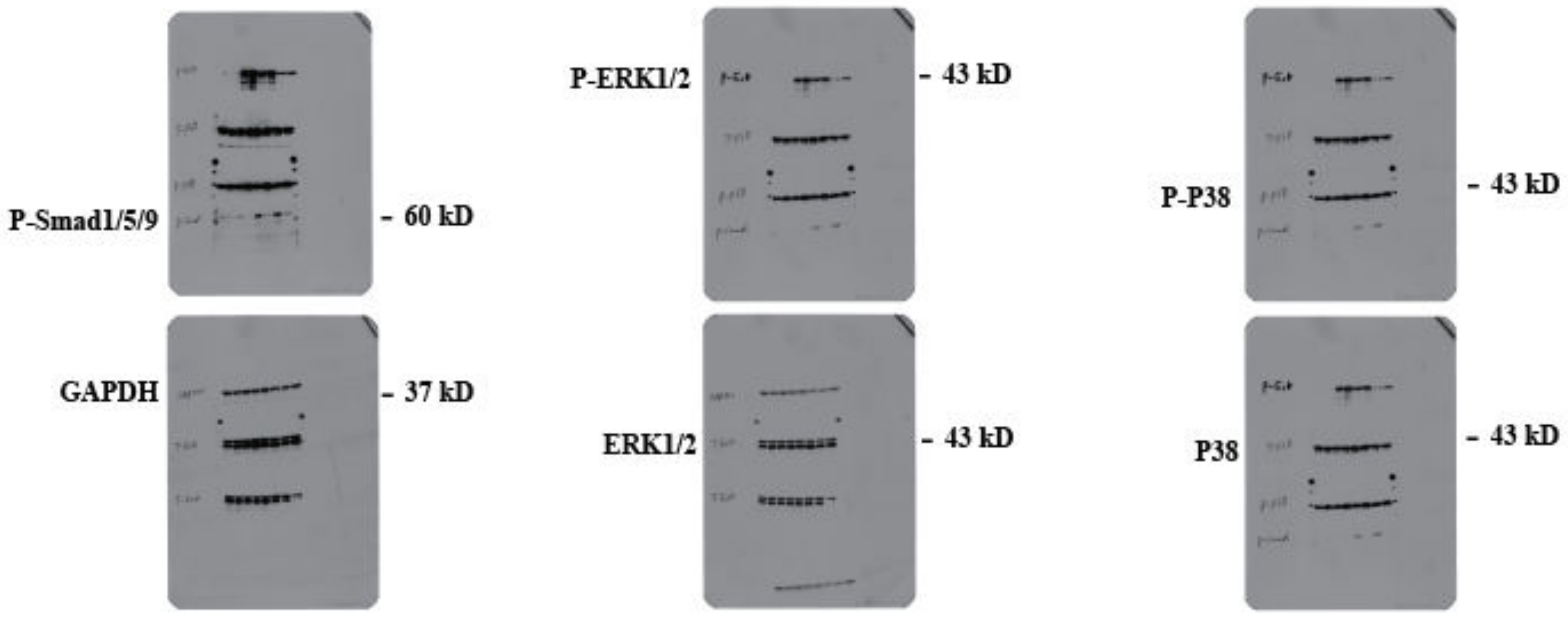

Fig. 6E

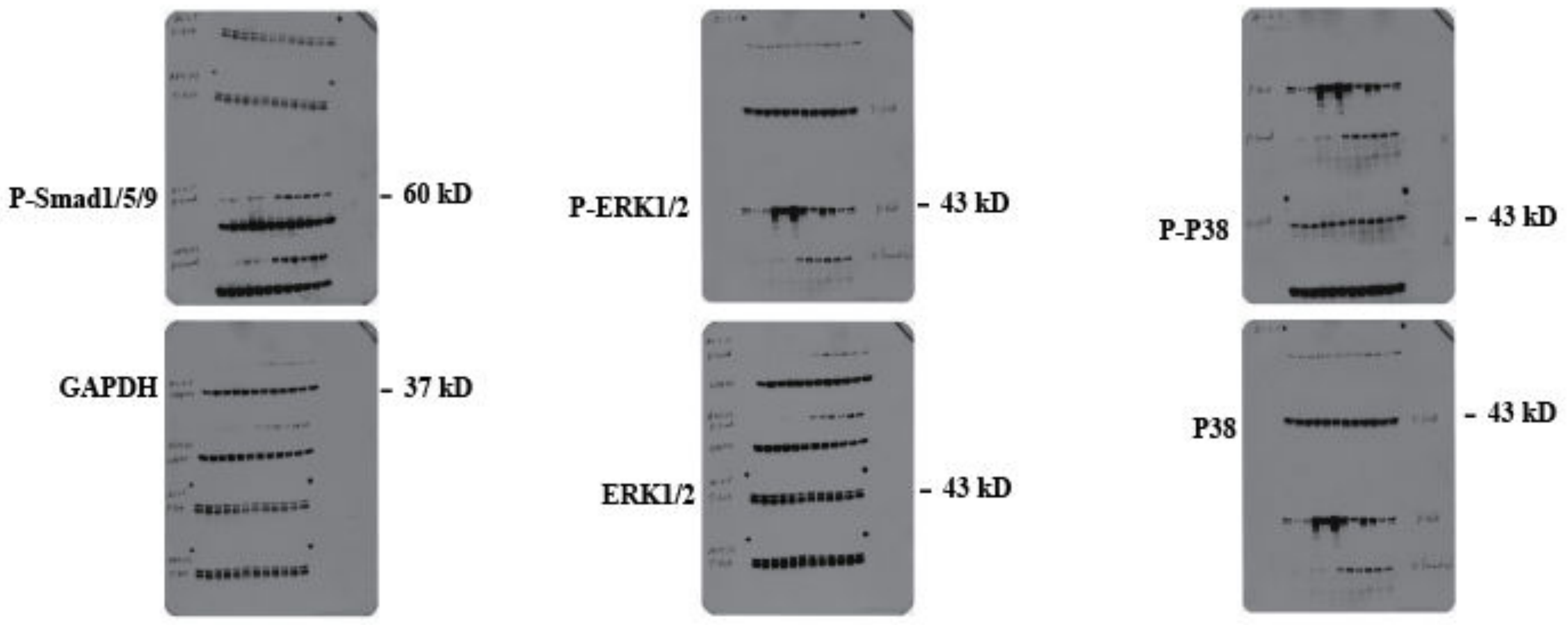

Shi, *et al.*, Supplementary Figure S13
